# Machine Learning Techniques for Simulating Human Psychophysical Testing of Low‐Resolution Phosphene Face Images in Artificial Vision

**DOI:** 10.1002/advs.202405789

**Published:** 2025-02-22

**Authors:** Na Min An, Hyeonhee Roh, Sein Kim, Jae Hun Kim, Maesoon Im

**Affiliations:** ^1^ Brain Science Institute Korea Institute of Science and Technology (KIST) Seoul 02792 Republic of Korea; ^2^ Sensor System Research Center Advanced Materials and Systems Research Division KIST Seoul 02792 Republic of Korea; ^3^ Division of Bio‐Medical Science and Technology University of Science and Technology (UST) Seoul 02792 Republic of Korea; ^4^ KHU‐KIST Department of Converging Science and Technology Kyung Hee University Seoul 02447 Republic of Korea; ^5^ Present address: Kim Jaechul Graduate School of AI KAIST Seoul 02455 Republic of Korea

**Keywords:** artificial vision, human psychophysical test, machine learning, prosthetic vision

## Abstract

To evaluate the quality of artificial visual percepts generated by emerging methodologies, researchers often rely on labor‐intensive and tedious human psychophysical experiments. These experiments necessitate repeated iterations upon any major/minor modifications in the hardware/software configurations. Here, the capacity of standard machine learning (ML) models is investigated to accurately replicate quaternary match‐to‐sample tasks using low‐resolution facial images represented by arrays of phosphenes as input stimuli. Initially, the performance of the ML models trained to approximate innate human facial recognition abilities across a dataset comprising 3600 phosphene images of human faces is analyzed. Subsequently, due to the time constraints and the potential for subject fatigue, the psychophysical test is limited to presenting only 720 low‐resolution phosphene images to 36 human subjects. Notably, the superior model adeptly mirrors the behavioral trend of human subjects, offering precise predictions for 8 out of 9 phosphene quality levels on the overlapping test queries. Subsequently, human recognition performances for untested phosphene images are predicted, streamlining the process and minimizing the need for additional psychophysical tests. The findings underscore the transformative potential of ML in reshaping the research paradigm of visual prosthetics, facilitating the expedited advancement of prostheses.

## Graphical abstract

To evaluate the quality of artificial visual percepts generated by emerging methodologies, researchers often rely on labor‐intensive and tedious human psychophysical experiments. The present work investigates the capacity of standard machine learning models to accurately replicate quaternary match‐to‐sample tasks using low‐resolution facial images represented by arrays of phosphenes as input stimuli for facilitating the expedited advancement of visual prostheses.

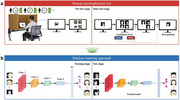

## 1 Introduction

It has been widely demonstrated that the behavioral patterns of machine learning (ML) models and humans are comparable when recognizing objects presented in high‐resolution images^[^ ^1^, ^2^, ^3^, ^4^, ^5^, ^6^, ^7^ ^]^ The efficacy and speed of testing procedures of these ML models significantly surpass the time‐consuming and laborious nature of human psychophysical experiments,^[^ ^8^ ^]^ particularly due to the readily available nature of publicly trained ML models for inference tasks. While these ML models were initially trained on large‐scale datasets with extensive computational resources, their testing phase can be performed more efficiently in comparison to human experiments. Consequently, a substantial body of literature exists that compares the behavioral responses of humans versus machines, not only for original images but also for those that are noisy or corrupted^[^ ^1^, ^2^, ^9^, ^10^, ^11^, ^12^, ^13^, ^14^ ^]^ ML models. Particularly, convolutional neural networks (CNNs) have exhibited remarkable capabilities across diverse image recognition tasks,^[^ ^15^, ^16^, ^17^ ^]^ suggesting the potential to replicate human performance even with non‐natural visual stimuli such as distorted,^[^ ^9^ ^]^ noisy,^[^ ^10^ ^]^ or low‐resolution representations.

In the domain of visual prosthetics, ML models also hold substantial promise for evaluating the quality of artificial vision,^[^ ^18^, ^19^, ^20^, ^21^, ^22^, ^23^, ^24^, ^25^, ^26^, ^27^, ^28^, ^29^ ^]^ which often appears in low‐resolution forms. Currently, visual prostheses offer individuals with profound vision loss a rudimentary form of artificial vision^[^ ^30^ ^]^ composed of bright spots known as “*phosphenes*,” achieved through stimulation of the retina,^[^ ^31^, ^32^, ^33^, ^34^ ^]^ optic nerve,^[^ ^35^, ^36^ ^]^ lateral geniculate nucleus,^[^ ^37^, ^38^, ^39^ ^]^ or visual cortex.^[^ ^40^, ^41^, ^42^, ^43^ ^]^ Despite successful commercialization of retinal prosthetic systems^[^ ^44^, ^45^, ^46^ ^]^ and clinical trials employing electric^[^ ^47^, ^48^, ^49^, ^50^, ^51^ ^]^ or optogenetic^[^ ^52^ ^]^ approaches, prosthetic users have not yet attained vision levels surpassing 20/200 vision or the threshold of legal blindness (but note that better visual acuity was achieved recently with electronic magnification^[^ ^53^ ^]^). Moreover, the elicited visual percepts (i.e., phosphenes) vary significantly across subjects,^[^ ^54^ ^]^ necessitating tailored stimulation parameters (e.g., frequency, amplitude, pulse duration) for each implant recipient.^[^ ^55^, ^56^, ^57^, ^58^, ^59^, ^60^ ^]^ To improve clinical outcomes in prosthetic users, numerous research groups have investigated new hardware/software components, including electrode layouts,^[^ ^36^, ^50^, ^61^, ^62^ ^]^ stimulation strategies,^[^ ^51^, ^63^, ^64^, ^65^, ^66^, ^67^, ^68^, ^69^ ^]^ and phosphene configurations.^[^ ^18^, ^21^, ^22^, ^23^, ^25^, ^55^, ^56^, ^58^, ^70^, ^71^, ^72^ ^]^ Additionally, to identify optimal design parameters that enhance the quality of visual percepts represented with the limited number of pixels and grayscale levels generated by visual prostheses, it is essential to characterize the behaviors of the implant users^[^ ^31^, ^32^, ^33^, ^34^, ^35^, ^36^, ^37^, ^39^, ^40^, ^44^, ^45^, ^46^, ^47^, ^48^, ^49^, ^50^, ^51^, ^52^, ^54^, ^55^, ^56^, ^57^, ^68^, ^69^, ^73^ ^]^ or sighted individuals with simulated artificial vision.^[^ ^18^, ^21^, ^22^, ^23^, ^25^, ^27^, ^58^, ^70^, ^71^, ^72^ ^]^ This conventional approach remains indispensable for advancing the development of visual prosthetic technologies.

**Figure 1:**
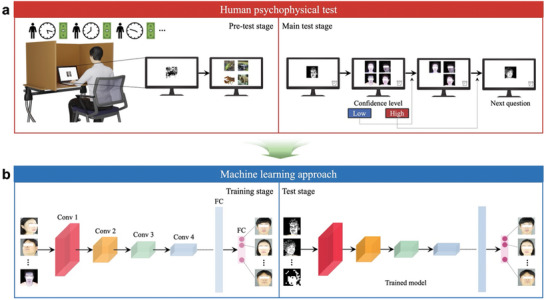
Schematic diagrams of human psychophysical testing and machine learning (ML) approaches for measuring face recognition accuracy in phosphene images for artificial vision research. a) Human psychophysical tests comprised Pretest and Main test stages, conventionally employed to assess the effectiveness of visual prostheses by simulating artificial vision with low‐resolution phosphene images on computer screens. The Pretest stage familiarized human participants with the experimental procedure using nonhuman faces displayed on the screens. Subsequently, human faces were presented in the Main test session to evaluate the phosphene facial recognition accuracies. Participants were given the option to reconsider their initial answer choice if they lacked confidence, allowing them to choose the next best option on the subsequent screen. b) We explored whether trained ML models could aid human psychophysical tests by emulating the innate facial recognition ability of humans using two stimulation types (e.g., Non‐Gaussian‐Blurred; NGB and Gaussian‐Blurred; GB versions). The ML models classify different facial identities using a fully connected (FC) layer, where each artificial neuron (magenta circle) corresponds to the probability of one facial class. The number of convolution (Conv) layers and other types of layers differ across ML models (*see* Figure S8, Supporting Information, for details). This research used datasets from 'The Open AI Dataset Project (AI‐Hub, S. Korea)' with explicit permission from the original creators. All data information can be accessed through 'AI‐Hub (www.aihub.or.kr)' (Choi et al., *arXiv*, 2021).^[^ ^123^ ^]^ Note that the eyes of the high‐resolution facial images are covered for privacy.

Generally, conducting psychophysical tests on human subjects is a considerably time‐consuming and labor‐intensive endeavor (**Figure** **1**a). Planning the experimental procedure alone typically spans several months, including Institutional Review Board (IRB) approval, which consumes three to four months on average^[^ ^74^ ^]^ While it may be ideal for tests to encompass a wide array of stimulation parameter combinations (e.g., pixel size and grayscale level related to frequency and/or amplitude),^[^ ^18^, ^21^, ^23^, ^25^, ^51^, ^55^, ^56^, ^57^, ^70^, ^71^, ^72^, ^73^ ^]^ scene simplification strategies (e.g., segmentation,^[^ ^22^, ^27^, ^58^ ^]^ saliency,^[^ ^58^ ^]^ and depth,^[^ ^58^ ^]^) and image conditions (e.g., object size,^[^ ^1^, ^2^, ^75^ ^]^ viewpoint angle,^[^ ^1^, ^2^, ^26^, ^75^ ^]^ lighting level,^[^ ^75^ ^]^ and noise type^[^ ^1^, ^2^, ^9^, ^12^, ^13^ ^]^), collecting large‐scale behavioral data remains challenging due to constraints such as participant fatigue and muscle pain^[^ ^9^ ^]^ Moreover, any minor adjustments to design parameters necessitate additional extended sets of psychophysical tests. Despite significant technological advancements in artificial vision,^[^ ^31^, ^32^, ^33^, ^34^, ^35^, ^36^, ^37^, ^39^, ^40^, ^44^, ^45^, ^46^, ^47^, ^48^, ^49^, ^50^, ^51^, ^52^, ^54^, ^55^, ^56^, ^57^, ^68^, ^69^, ^73^ ^]^ improving the efficiency of psychophysical tests remains elusive, leaving a bottleneck in the typically lengthy turnaround time of visual prosthetic development.

Considering that ML has demonstrated greater time‐ and cost‐efficiency compared to human subjects in various tasks,^[^ ^4^, ^76^ ^]^ it holds the potential to assist in sparing development time, thereby opening up more opportunities for breakthroughs in visual prostheses. However, even with recent efforts,^[^ ^28^, ^58^, ^77^, ^78^ ^]^ the application of ML in developing prosthetic systems still remains incomplete.^[^ ^19^, ^79^ ^]^ Among various ML models, deep learning (DL) models, in particular, are known to resemble the different areas of the ventral stream (V1, V2, V4, and inferior temporal (IT) cortex).^[^ ^1^ ^]^ These correspondences can be captured with high Brain‐Score,^[^ ^5^, ^80^ ^]^ a comprehensive benchmark that assesses how much computational models align with the neural and behavioral data, with their own “artificial neurons” corresponding to functional neurons of the primate visual system.^[^ ^5^, ^81^, ^82^, ^83^ ^]^ Consequently, these artificial models may provide an effective alternative to costly psychophysical testing of prosthetic vision (Figure 1b). However, prior ML studies have not tested downsampled artificial vision expressed in arrays of phosphenes, further complicating object recognition. Additionally, no comparative studies have constructed a practical model based on the highly correlated relationship between humans and machines, and no attempt has been made to shift a traditional paradigm of solely accumulating behavioral trials from humans to using ML models for psychophysical tests of artificial vision.

In the present study, we explored whether ML could facilitate human psychophysical experiments using phosphene images simulating artificial vision (Figure 1). Since facial recognition has been paramount as a socio‐cognitive function since birth and is crucial in daily living,^[^ ^75^, ^82^, ^84^, ^85^, ^86^, ^87^ ^]^ we tested the ML models on *facial* phosphene images. Specifically, we focused on datasets containing only facial images with a plain background to eliminate shape‐based recognition cues across various objects^[^ ^1^, ^2^, ^10^ ^]^ and ensured that both humans and machines relied solely on facial features for identification. We began by comparing twelve ML models to identify the model with the highest facial recognition accuracy (the best model) across various resolutions and grayscale (GS) levels, referred to hereafter as phosphene image quality (PIQ) levels. Then, to quantify how closely the best ML model mirrors human behavioral patterns, we performed psychophysical testing with human subjects using a subset of visual stimuli previously tested on the ML models. Based on these ML and human experimental results, we demonstrated the possibility of estimating human recognition performances on untested facial phosphene images using our proposed experimental design with linear and nonlinear models trained on the relationships between the performances of humans and machines for the tested images.

Furthermore, to delve deeper into recognition performances at the image level, we disentangled the average recognition accuracy of faces within a given PIQ level into accuracy per each facial image. This image‐level analysis revealed a discrepancy between the ML models and the humans, underscoring the need for further research to achieve a complete replacement of psychophysical testing. Additionally, to offer insights for potential algorithms better mimicking human facial recognition, we visualized the Grad‐CAM of the models and compared them with eye‐tracking heatmaps of the human subjects, unveiling distinct regions of interest for decision‐making of the two systems. In summary, the present study suggests that employing ML models can substantially reduce both the time and cost required for human psychophysical tests, thereby expediting the overall turnaround time for developing new visual prosthetic systems by shortening investigations into their expected efficacies.

## 2 Results

### 2.1 Facial Recognition Accuracies are Similar Between Human Averages and The Best ML Model Across Varying PIQ Levels

Before comparing the recognition accuracies of normally sighted human subjects and ML models for low‐resolution face images, we first examined the performances of twelve ML models, including end‐to‐end DL models (Figure S1a, Supporting Information), using high‐resolution grayscale face images. The first two models (M01 and M02) were selected as the baseline; unlike the other models, these two models utilized only pixel values without extracting image features to classify facial classes. This approach aligns with previous work.^[^ ^2^ ^]^ Models M03 and M04 were widely‐used ML models, where image features were extracted using principal component analysis.^[^ ^88^ ^]^ The last eight models were DL models trained by the authors from scratch, including convolutional neural network (CNN; M05 and M06), AlexNet^[^ ^15^ ^]^ (M07 and M08), visual geometry group (VGG^[^ ^17^ ^]^; M09 and M10), and residual neural network (ResNet^[^ ^89^ ^]^; M11 and M12). These models were selected for their broad applicability as CNN‐based DL models in the AI field (see the Experimental Section for training details). It was not surprising that all ML models we tested (M01‐M12; *see* Materials and Methods) demonstrated recognition accuracies exceeding 80%. Particularly, all DL models (M05‐M12; dark blue bars of Figure S1b in the Supporting Information) achieved near‐human level performance (i.e., almost 100%) and outperformed conventional ML models (light blue bars of Figure S1b, Supporting Information). Notably, both non‐DL and DL groups exhibited comparable performances across the models in each group (i.e., sky blue vs dark blue bars in Figure S1 in the Supporting Information).

**Figure 2:**
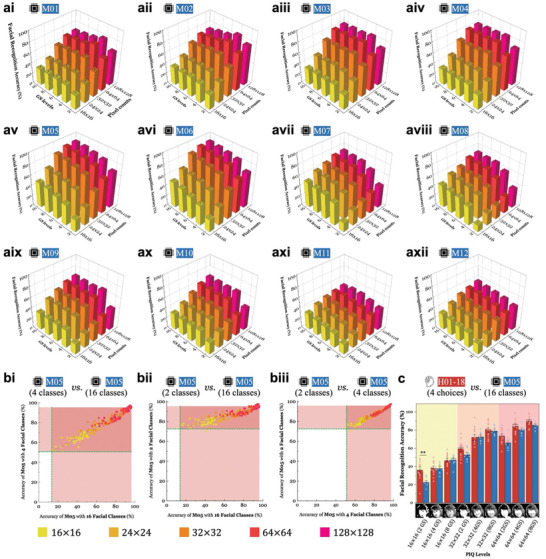
For the phosphene images in non‐Gaussian‐blurred (NGB) version, the facial recognition accuracy of machine learning (ML) models closely mirrors that of humans. ai‐axii) Twelve ML models were tested to determine their ability to recognize correctly faces out of 16 facial classes across 5 resolutions and 5 grayscale levels (referred to as phosphene image quality; PIQ levels). For most models, resolution levels (i.e., pixel counts) had a more significant impact on facial recognition accuracy compared to grayscale levels. Model M05, identified as the Best Model, consistently outperformed other models across nearly all PIQ levels. Error bars in each panel represent the standard errors of 16 facial classes and 9 facial conditions. (bi‐biii) The Best Model exhibited a larger range (band gap) of accuracy when tested with a relatively greater number of facial classes (e.g., 16) compared to a small number of facial classes (e.g., 2 or 4). Green dotted lines indicate the minimum recognition accuracy for the lowest‐resolution images for each given number of facial classes. However, high linear correlations exist across 25 PIQ levels between M05s tested with two different numbers of facial classes as follows: (bi) 16 and 4 (*r* = 0.96, *n* = 225, *p* < 0.001) bii) 16 and 2 (*r* = 0.90, *n* = 225, *p* < 0.001), and biii) 4 and 2 (*r* = 0.97, *n* = 225, *p* < 0.001). Therefore, the performance order remained consistent even with modifications to the number of artificial neurons in the last layer. c) The facial recognition accuracy of M05 with a facial class size of 16 closely matched human performances across 8 out of 9 (89%) PIQ levels. Each bar indicates the average and one standard deviation of 18 human subjects and 10 model instances (i.e., independent runs) (^*^ *p* < 0.05, ^**^ *p* < 0.01, ^***^ *p* < 0.001, Wilcoxon rank sum test). Human brain icon was obtained from Flaticon.com. Note that the eyes of the high‐resolution facial images are covered for privacy.

In sharp contrast, however, each individual model exhibited markedly different patterns for low‐resolution face images expressed in arrays of phosphenes across varying PIQ levels (**Figures** **2** and S2 in the Supporting Information for non‐Gaussian‐blurred, NGB for short, and Gaussian‐blurred, GB for short, phosphenes, respectively; see the Experimental Section). When we tested a total of 3600 phosphene images (comprising 25 PIQ levels, 16 facial classes, and 9 facial conditions), all models generally performed poorly for low PIQ levels, but their performances varied significantly across models (e.g., *compare* Figures 2av vs 2axi). Moreover, as resolution decreased, most ML models exhibited more pronounced drops in recognition accuracies with GB phosphenes compared to NGB phosphenes in low resolutions (*compare* yellow bars in Figures S2ai‐axii, Supporting Information vs those of Figures S2ai‐axii in the Supporting Information). These findings suggest that each ML model possesses considerably different capabilities for recognizing faces, which are thought to be presented by visual prosthetic systems depending on stimulation methodologies (i.e., optogenetic vs electric stimulation for NGB vs GB phosphenes; *see* the Experimental Section). However, it should be noted that this does not imply a direct one‐to‐one correspondence between model performance and implant technologies. Other unexplored ML models might better align with the tested stimulation strategies, and further investigations are necessary to explore these possibilities.

Although all models encountered significant challenges with face images in poor PIQ levels, they demonstrated an increasing trend in facial recognition accuracy as either pixel counts or grayscale (GS) levels increased, consistent with previous studies^[^ ^25^ ^]^ Notably, the effect of pixel counts was larger than that of the GS levels (Figure 2ai‐axii and Figure S2ai‐axii, Supporting Information; Figure S1ci‐cv and S1di‐dv, Supporting Information, for 2D version of Figures 2ai‐2axii). Interestingly, for both training loss functions we used (i.e., cross‐entropy and multimargin losses), the shallowest DL model (M05/M06) outperformed the baseline model (M01/M02) and other DL models (M07/M08, M09/M10, and M11/M12) across every PIQ level, irrespective of phosphene types (i.e., NGB or GB). This suggests that *deeper* models may not always be the best option for processing the low‐resolution phosphene images, which have far less information than the original high‐resolution images. Also, M05/M06 outperformed M03/M04 when tested with NGB phosphenes (*compare* Figures 2aiii‐2aiv vs 2av‐2avi), although this was not the case with GB phosphenes in resolutions below 64 × 64 (*compare* Figure S2aiii‐aiv vs S2av‐avi, Supporting Information). Taken together, M05 and M06 exhibited the best facial recognition performances for the NGB phosphene images. Due to using a more common loss function, we will refer to M05 as the Best ML Model (or simply the Best Model) throughout this study. It should be noted that we set the Best Model based on the performance of the ML models tested on NGB phosphenes rather than GB phosphenes to investigate the effect of different types of phosphenes (i.e., NGB or GB) on the same model.

We then wondered whether the performance trend of the Best Model remains invariant across different numbers of neurons in the final layer. In the abovementioned test, 16 facial classes were given to the models as options for selection by incorporating 16 artificial neurons in the last layer of each model. Apparently, a smaller number of neurons would result in fewer options in the model, potentially reducing confusion and thereby enhancing accuracies. To explore this, we additionally tested with 2 and 4 neurons in the final layer of the model and examined the accuracy changes compared with the 16‐neuron cases. Indeed, the minimum accuracy was improved significantly (*compare* the shades projected on the *y*‐ and *x*‐axes in Figure 2bi for 4 vs 16 classes, Figure 2bii for 2 vs 16 classes, and Figure 2biii for 2 vs 4 classes; similar differences in recognition accuracy were observed in Figure S2bi‐biii, Supporting Information for the GB versions).

More importantly, it is worth noting that the performances of the Best Model with the two different numbers of facial classes showed highly linear correlations (Pearson's *r* = 0.96, 0.90, and 0.97 for Figures 2bi, 2bii and 2biii, respectively for NGB versions; *r* = 0.96, 0.90, and 0.97 for Figure S2bi‐biii, Supporting Information, respectively, for GB versions; *n* = 25 PIQ levels × 9 facial conditions), implying that the overall rank of performances depending on PIQ‐level is invariant even when we changed the number of artificial neurons in the final layer of the Best Model. This is a critical feature for efficiently estimating facial recognition accuracies using ML models. It is because if all 16 facial classes were tested with fewer neurons in the last layer, computational expenses would increase significantly, with 16 combinations 2 (= 120) times or 16 combinations 4 (= 1820) times higher for 2 or 4 neurons, respectively. Therefore, to maximize the computational efficiency, we maintained the layer with 16 neurons (or facial classes).

After identifying the Best Model (i.e., M05) from ML model experiments and choosing the reasonable value for the number of facial classes (i.e., 16), we selected 720 primary test images (20% of the total 3600 test images) for display in the human psychophysical test of artificial vision (*see* the Supporting Information for details). We compared the recognition performance of the Best Model with 16 facial classes and the innate facial recognition ability of humans in recognizing the correct face from 4 facial classes (i.e., 4 multiple choices) pooled from the same 16 facial classes tested with the Best Model (*see* Experimental Section). This approach is similar to an earlier work that compared 20 object classes for models versus binary object discrimination tasks for humans.^[^ ^2^ ^]^

In general, the facial recognition accuracy trend of the Best Model across varying PIQ levels followed that of the human subjects (Figure 2c and Figure S2c, Supporting Information). It was remarkably surprising that there were almost no statistically significant differences in the facial recognition performances of the Best Model with the human average in 8 out of 9 (≈89%) PIQ levels we tested for the NGB version (Figure 2c). The human subjects outperformed the machines only for images shown in 16 × 16 phosphenes with 2 GS (*p* < 0.01). However, the performance gap between the Best Model and the human average became pronounced when tested with GB phosphenes, especially for lower resolutions (e.g., 16 × 16) (*se*e bar graphs in yellow shades in Figure 2c and Figure S2c, Supporting Information). The same Best Model was not able to recognize faces up until a resolution level of 32 × 32 as accurately as humans (Figure S2c, Supporting Information; *p* < 0.01 for all but 32 × 32 with 8 GS, which showed *p* < 0.05), implying that the Best Model presented in this paper could serve as a baseline model for building ML models that are robust in recognizing different types of low‐resolution phosphene images to catch up with the human visual system^[^ ^9^, ^10^, ^12^, ^13^, ^14^ ^]^ (*see* Discussions). In addition, the logarithmic trend of the facial recognition ability across PIQ levels for both humans and ML models aligns with previous findings^[^ ^90^, ^91^ ^]^ that merely increasing the density of electrodes or the number of “pixels” may not significantly improve perception (i.e., facial recognition) ability.

### 2.2 The Correlation Between the Performances Of Human Subjects and the ML Model can be Utilized to Predict Human Performances at Untested PIQ Levels

**Figure 3:**
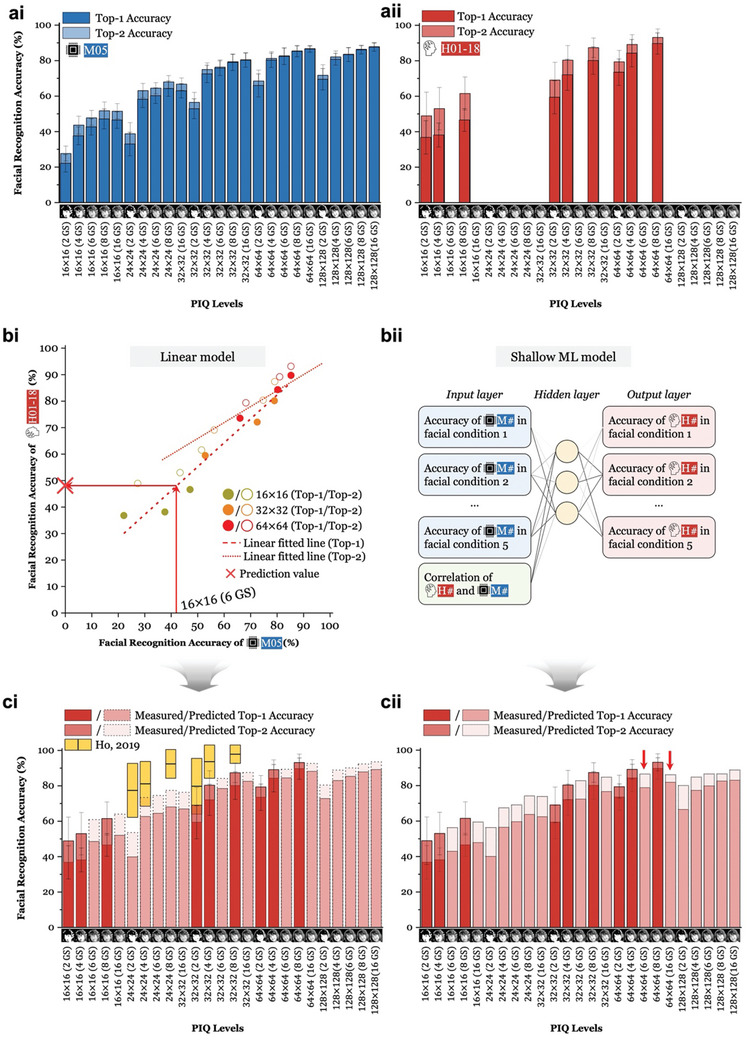
Prediction of human performances at unmeasured phosphene image quality (PIQ) levels using the strong correlation between the recognition performances of humans and machine learning (ML) models for Non‐Gaussian‐Blurred (NGB) version phosphene face images. Top‐1 and Top‐2 facial recognition accuracies of ai) the Best Model (i.e., M05) and aii) human participants. Each bar represents the average and one standard deviation of 80 (16 facial classes × 5 facial conditions) and 144 (16 facial classes × 9 facial conditions) test images for the humans and the M05, respectively. The human subjects (*n* = 18) were tested for only 9 PIQ levels, while the Best Model instances (*n* = 10) were tested for all 25 PIQ levels. The Best Model accuracies were used to predict the remaining 16 PIQ levels in human psychophysical testing, totaling 2,880 test images (16 PIQ levels × 16 facial classes × 9 facial conditions). bi) Given the comparable performance trends observed between the averages of 18 human subjects and 10 model instances across the nine tested PIQ levels for both Top‐1 (*r* = 0.97, *p* < 0.001) and Top‐2 accuracies (*r* = 0.88, *p* < 0.01), a linear regression was applied to the data. The resulting linear fitting curves are depicted by a dashed red line for Top‐1 accuracy and a dotted red line for Top‐2 accuracy, facilitating the estimation of human accuracy values at unmeasured PIQ levels (e.g., 16 × 16 with 6 grayscale (GS) levels). bii) Additionally, a nonlinear model, specifically the shallow ML model, was utilized to characterize the relationship between the Best Model instances and the humans using the accuracy of each human subject/model instance and their correlation levels. Subsequently, face recognition accuracies of humans for the unmeasured PIQ levels were estimated using both linear and nonlinear methodologies, respectively. ci) Across several PIQ levels, the predicted values of human performances generated by the linear model approximately followed the trend of recognition accuracies reported in a previous study (Ho et al., *J. Vis*., 2019).^[^ ^25^ ^]^ Tangerine bars represent the mean (indicated by black lines in the middle) and standard deviations. cii) The nonlinear model produced several estimation values that were notably lower than neighboring measured values, indicating that the linear model might suffice for predicting performances unmeasured in psychophysical testing. Human brain icon was obtained from Flaticon.com.

Using the Best Model selected from the previous section, we observed a strong correlation between its performance and that of the human subjects. This finding highlights the potential to streamline human psychophysical testing by leveraging ML models, such as the Best Model in our case, to estimate facial recognition accuracies for untested conditions. For all PIQ levels tested with the Best Model, we plotted bar graphs depicting Top‐1 and Top‐2 accuracies for both the model and the humans (**Figures** **3**ai‐aii and S3ai‐aii (Supporting Information) for NGB and GB phosphenes, respectively). Top‐1 accuracies represent the face recognition accuracies obtained when considering the first facial classes chosen by humans and the maximum probability facial classes computed by the model, corresponding to the accuracies shown in Figures 2 and Figure S2 (Supporting Information). On the other hand, Top‐2 accuracies represent those when considering both the first and second selections made by humans and the two facial classes with the highest probabilities computed by the model. Note that out of 3600 test images corresponding to 25 PIQ levels, 16 facial classes, and 9 facial conditions, which were tested using the Best Model (Figure 3ai and Figure S3ai, Supporting Information), the subsampled 720 test images (= 9 PIQ levels × 16 facial classes × 5 facial classes) were presented in the psychophysical testing (Figure 3aii and Figure S3aii, Supporting Information). We wondered whether we could estimate the Best Model performances for the remaining 2880 test images corresponding to 16 PIQ levels, exploiting the strong correlation between the Best Model and human average on tested 720 images to save tremendous human labor and costs that can be incurred from the notorious psychophysical testing.

Initially, we employed linear regression to fit the tested human data and the corresponding performances of the Best Model (M05) for both Top‐1 and Top‐2 accuracies (Figure 3bi and Figure S3bi, Supporting Information, for NGB and GB phosphenes, respectively). The robust linear relationship between humans and the Best Model across measured PIQ levels for both Top‐1 (*r* = 0.97 and 0.96 for NGB and GB phosphenes) and Top‐2 accuracies (*r* = 0.88 and 0.96 for NGB and GB phosphenes) allowed us to utilize these linear fitting curves to predict human recognition performances for untested PIQ level conditions (Figure 3ci and Figure S3ci, Supporting Information, for NGB and GB phosphenes, respectively). To show that this linear model is sufficient to model the relationship between the performances of human average and the Best Model, we also built and trained a one‐layered DL model, denoted as a shallow ML model, using 1620 trial data (*see* Materials and Methods for detail) containing information on ML and human performances for facial recognition in low‐resolution phosphene images (Figure 3bii and Figure S3bii, Supporting Information, for NGB and GB phosphenes, respectively; *see* Materials and Methods). We found that several estimates derived from the nonlinear model results (pointed out by red arrows in Figure 3cii and Figure S3cii, Supporting Information) were lower compared to neighboring performances, particularly for GB phosphenes (*compare* Figure S3ci,cii, Supporting Information), justifying our initial choice of opting for the linear model when estimating human behavioral trend across unmeasured PIQ levels compared to the shallow ML model.

Importantly, our estimated performances obtained from the linear model exhibited an increasing trend of facial recognition accuracy proportional to the number of pixels and grayscale level, consistent with previous findings^[^ ^21^, ^25^ ^]^ (Figure 3ci and Figure S3ci, Supporting Information, for NGB and GB phosphenes, respectively). Nevertheless, our psychophysical/estimated recognition accuracies did not align precisely with those reported by similar previous works,^[^ ^18^, ^21^, ^25^ ^]^ probably due to differences in experimental conditions such as allowed scanning time and source of face images. For instance, the performance disparities between the past work^[^ ^25^ ^]^ and our NGB version (Figure 3ci) may stem from the longer scanning duration exceeding five seconds in the previous study, whereas we imposed a three‐second time limit per trial to accommodate the assessment of various parameters within a constrained timeframe. Additionally, the performance estimates were slightly lower than those reported in the previous research^[^ ^21^ ^]^ (Figure S3ci, Supporting Information), potentially due to the absence of a matching working environment between our subjects and the individuals featured on the test screens. Consequently, after identifying an ML model demonstrating superior performance across various types of stimuli (e.g., GB and NGB), a linear model may suffice to predict performances for untested PIQ levels in psychophysical testing.

### 2.3 Image‐by‐Image Analyses Disclose Humans and ML Models Exhibit Different Face‐Dependent Discrimination Abilities

**Figure 4:**
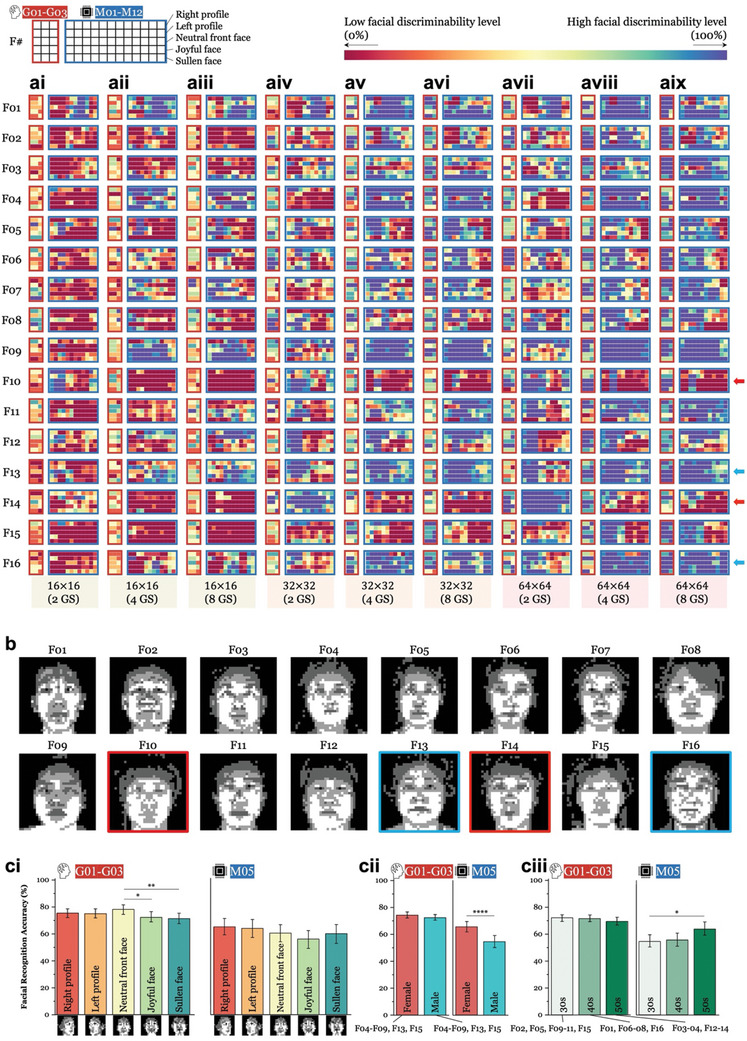
Differences in image‐level facial discriminability between humans and machine learning (ML) models for non‐Gaussian‐blurred (NGB) version phosphene images. ai‐aix) Both human groups (G01‐G03) and ML models (M01‐M12) struggled to recognize most of the test images at the lower resolution (i.e., 16 × 16), as evidenced by the prevalence of warm‐colored bins. However, the human groups exhibited progressively better performance as the phosphene image quality (PIQ) level increased, with few or no red squares at the resolution level of 64 × 64. In contrast, the ML models still occasionally showed red squares at higher resolutions (i.e., 64 × 64). From the PIQ level of 32 × 32 (4 grayscales, or GS) to the highest PIQ level, most ML models encountered challenges in differentiating two facial classes (F10 and F14 indicated by red arrows shown on the right) while demonstrating decent discrimination for two other facial classes (F13 and F16 indicated by blue arrows). b) All 16 facial classes (F01‐F16) are shown here at the PIQ level of 32 × 32 (4 GS). F10 and F14 are outlined with red boxes, indicating facial classes that posed challenges even at the highest PIQ level, while F13 and F16 are outlined with blue boxes, signifying facial classes were easily discriminated. ci‐ciii) We examined the effects of facial conditions, gender, and age on the performance of the human groups (i.e., G01‐03) and the Best Model (i.e., M05). While the human subjects exhibited varied performance across different facial expressions, the Best Model displayed nearly uniform performance across those expressions (*ci*). Conversely, while the humans demonstrated consistent performance regardless of gender (*cii*) and age (*ciii*), the Best Model exhibited biases in both gender and age, showing statistically better performances for recognizing female and older faces expressed by NGB phosphenes. Each bar represents the average and one standard deviation of 144 (*ci*), 360 (*cii*), and 225‐270 (*ciii*) images, respectively (^*^ *p* < 0.05, ^**^ *p* < 0.01, ^***^ *p* < 0.001; Wilcoxon rank sum test). This research used datasets from 'The Open AI Dataset Project (AI‐Hub, S. Korea)' with explicit permission from the original creators. All data information can be accessed through 'AI‐Hub (www.aihub.or.kr)' (Choi et al., *arXiv*, 2021).^[^ ^123^ ^]^ Human brain icon was obtained from Flaticon.com.

Typically, humans do not exhibit varying levels of difficulty in recognizing specific faces. To explore whether ML models encounter challenges in distinguishing particular face(s), we disaggregated the average facial recognition accuracies into accuracies for each facial class (F01‐F16) and each facial condition (right, light, neutral front, joyful, and sullen faces; *see* top left legend in **Figures** **4** and Figure S4, Supporting Information) for both every human group and model (Figure 4ai and Figure S4ai, Supporting Information). For the 36 human subjects tested in our study, every three subjects were grouped together based on the type of question set that they were presented with (G01‐G03 and G04‐G06 were tested for NGB and GB images, respectively; *see* the Experimental Section), while each ML model (M01‐M12) was individually analyzed. This image‐by‐image analysis was motivated by the Brain‐Score behavioral benchmark,^[^ ^5^, ^80^ ^]^ which tested the similarity between human/monkey behavioral responses on binary object recognition tasks using natural images.^[^ ^2^ ^]^ Here, we extended this benchmark by providing image‐by‐image analysis of facial recognition ability on quaternary tasks using low‐resolution phosphene images. Overall, we found that even our Best Model did not successfully score high on an image level, similar to the past findings,^[^ ^2^ ^]^ highlighting the necessity of careful model selection/development.

Both humans (first 3 columns outlined with red boxes) and machines (following 12 columns outlined with blue boxes) exhibited low discriminability levels for particular faces in low‐resolution phosphene images formed with substantially fewer pixels (visible as frequent warm‐colored bins in Figure 4ai‐aiii and Figure S4ai‐aiii, Supporting Information, for NGB and GB phosphenes, respectively). For instance, at the PIQ level of 16 × 16 (2 GS), both human groups and ML models generally demonstrated less than 50% facial discriminability levels for the facial class F15. However, while the human group performances improved from the PIQ level of 32 × 32 (2 GS), the models appeared to have still low recognition ability (Figure 4a and Figure S4a, Supporting Information, for NGB and GB phosphenes, respectively; *see also* Figure 2c and Figure S2c, Supporting Information for the average results).

Although the ML models often exhibited at least 50% recognition accuracies (indicated by yellow bins) at the resolution level of 64 × 64, the test images of certain facial classes still proved challenging (indicated by red arrows; F10 and F14), while others were easier to be recognized (indicated by blue arrows; F13 and F16) for most ML models. For instance, at most PIQ levels, F10 and F14, which had relatively sharp chins, posed challenges for the models (faces outlined by red boxes in Figures 4b and Figure S4b, Supporting Information, for NGB and GB phosphenes, respectively). Conversely, F13 and F16, which had rounder chins, consistently exhibited higher facial discriminability levels than other facial classes (faces outlined by blue boxes in Figures 4b and S4b, Supporting Information for NGB and GB phosphenes, respectively). These results suggest that facial contours are interfering factors for ML models to discriminate faces in low‐resolution phosphene images compared to humans.

Additionally, we investigated whether facial conditions and demographics influenced the facial recognition accuracy of humans and the Best Model (M05), similar to findings of easy and difficult parameters (e.g., pose, lightening, expression) reported in previous studies.^[^ ^1^, ^2^, ^7^, ^92^ ^]^ We found that facial expressions affected human performances but not the model (Figures 4ci and S4ci, Supporting Information, for NGB and GB phosphenes, respectively), indicating the absence of facial condition bias, at least in the Best Model. In contrast, there were gender and age biases for M05 tested only with NGB, but not GB, phosphenes (*compare* Figure 4cii vs Figure S4cii, Supporting Information, for gender and Figure 4ciii vs Figure S4ciii, Supporting Information, for age). One possible explanation could be that M05 extracted more discernible information from female and older faces represented in NGB than GB phosphenes, possibly because NGB phosphenes rendered certain facial features (e.g., hair and fine lines) more prominent than GB phosphenes which are presented with more noise.^[^ ^93^ ^]^ Therefore, to enhance the resemblance of the Best Model to humans, it appears crucial to ensure that the model can accomplish comparably high cognitive performances at individual image levels and have reduced gender and age biases like humans.

### 2.4 ML Models and Humans Gather Key Information from Distinct Regions‐of‐Interest

**Figure 5:**
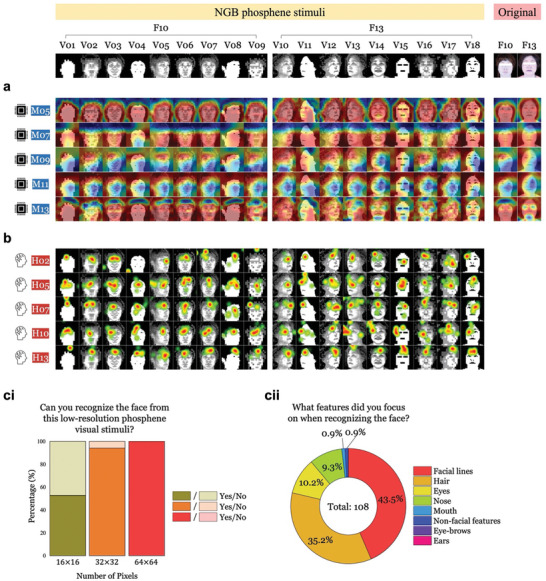
The region‐of‐interest for facial recognition differs between deep learning (DL) models and humans for non‐Gaussian‐blurred (NGB) version phosphene images. a) Gradient‐weighted Class Activation Maps (Grad‐CAMs) analysis was conducted for the four DL models (M05, M07, M09, and M11) along with one fine‐tuned DL model (M13), using 18 sample phosphene images representing two facial classes (i.e., V01‐V09 for F10 and V10‐V18 for F13). These samples covered various phosphene image quality (PIQ) levels and facial conditions. Notably, the Best Model (M05) consistently captured the facial features from the phosphene images better than the other models (M07, M09, M11, and M13), resembling the performance with the original high‐resolution images of those two facial classes (F10 and F13). This superior capability remained consistent across different PIQ levels, facial conditions, and facial classes (V01‐V18). b) The heat maps illustrating the relative duration of gaze area in five human subjects (H02, H05, H07, H10, and H13) were generated using Tobii Pro Fusion eye‐tracker data. Across the participants, there was a consistent tendency to gaze more at the upper parts of the faces, as indicated by the red regions. ci‐cii) During the Post‐test stage, survey responses were collected for each of the three different resolution levels with 4 grayscales (GS). First, 18 human subjects were asked if they recognized two faces represented with an array of phosphenes (36 responses in total for each resolution; 18 subjects × 2 facial classes). More than half of the subjects reported that they could not recognize faces from the 16 × 16 phosphenes, but the majority responded positively, starting from the 32 × 32 pixels (*ci*). Subsequently, those subjects were additionally asked about the visual features they had paid the most attention to (108 responses in total; 18 subjects × three resolutions × two facial classes). The human subjects had primarily focused on “facial lines” and “hair” among various facial features (*cii*). This research used datasets from 'The Open AI Dataset Project (AI‐Hub, S. Korea)' with explicit permission from the original creators. All data information can be accessed through 'AI‐Hub (www.aihub.or.kr)' (Choi et al., *arXiv*, 2021).^[^ ^123^ ^]^ Human brain icon was obtained from Flaticon.com. Note that the eyes of the high‐resolution facial images are covered for privacy.

To examine where the ML models and the human subjects primarily focused while attempting to recognize faces depicted in phosphene arrays, we utilized Gradient‐weighted Class Activation Maps (Grad‐CAMs)^[^ ^94^ ^]^ and eye‐tracking heatmaps, respectively (**Figure** **5**a,b for NGB, and Figure S5a,b, Supporting Information, for GB images). Since Grad‐CAMs are applicable only to DL models, we present here the results of DL models (M05, M07, M09, and M11) to shed light on the potential source(s) of performance discrepancies across the models. Additionally, we applied Grad‐CAMs to a fine‐tuned version of M05 (M13; *see* performance results of M13 in Figures S6ai‐aii, Supporting Information, for NGB and GB images, respectively) to illustrate the effect of phosphene images used in the training stage on the regions of interest for the models.

Notably, M05 consistently captured inner facial features with round shapes of contour lines for all sampled test images (the first rows in Figure 5a and Figure S5a, Supporting Information, for NGB and GB images, respectively). Also, it is worth noting that the Grad‐CAMs of M05 were quite consistent regardless of the order they were presented in the Main test stage, PIQ levels, and facial conditions and classes, which are similar to those for original (i.e., high‐resolution) face images (the right‐most two maps in Figure 5a). In contrast, the other models (i.e., M07, M09, M11, and M13) primarily focused on different visual stimuli areas during facial recognition (the second to the last rows in Figure 5a and Figure S5a, Supporting Information, for NGB and GB images, respectively), even for original high‐resolution test images as well. In particular, M07 and M09 constantly focused on the whole lower half side of the images and above the hair lines, respectively. Also, the fine‐tuned version of the Best Model (i.e., M13) focused on wider parts of the test images than M05, with a lower consistency of Grad‐CAMs across sampled test images than the other models. These results suggest that capturing inner facial features with facial contour lines is crucial for achieving the highest recognition performances among all ML models.

We further compared the Grad‐CAM results of the Best Model, M05, with the eye‐tracking results of the five human subjects (H02, H05, H07, H10, and H13 in Figure 5b for NGB phosphenes and H21, H24, H28, H31, and H34 in Figure S5b, Supporting Information, for GB phosphenes). Throughout the sampled phosphene images, the subjects consistently fixated their eyes on the upper parts of the faces near the hairline with a left gaze bias, which aligns with previous reports^[^ ^95^, ^96^ ^]^ (Figure 5b and Figure S5b, Supporting Information, for NGB and GB phosphenes, respectively). Intriguingly, this fixation pattern in humans was more similar to that observed in M09 (the ML model utilizing VGG architecture; the third rows in Figure 5a and Figure S5a, Supporting Information, for NGB and GB images, respectively) than to that observed in M05, suggesting that the Best Model (M05) may not extract key information from the most similar region‐of‐interest as humans. However, despite M09 showing more similar areas of interest with humans compared to M05, it is essential to note that the facial recognition accuracies for 64 × 64 resolution were only near 60% for 4, 6, 8, and 16 grayscales and just around 30% for 2 grayscale levels (Figures 2aix and S2aix, Supporting Information for NGB and GB images, respectively). These accuracies fall far below average human performances for the 64 × 64 resolution level (the red bars with the red background in Figure 2c and Figure S2c, Supporting Information, for NGB and GB images, respectively).

Although no differences were observed in the eye‐tracking results between NGB and GB phosphenes (*compare* Figure 5b vs Figure S5b, Supporting Information), the responses of human subjects to the first Post‐test multiple‐choice question indicated that more participants struggled to recognize faces expressed in GB than NGB phosphene images presented with 16 × 16 (4 GS) and 32 × 32 (4GS) phosphenes (*compare* percentages per each pixel count in Figure 5ci and Figure S5ci, Supporting Information). In addition, even though the Grad‐CAM results of the Best Model did not align with the eye‐tracking heatmaps (compare Figures 5a vs 5b and Figure S5a vs S5b, Supporting Information, for NGB and GB phosphenes, respectively), they matched the Post‐test multiple‐choice responses (Figure 5cii and Figure S5cii, Supporting Information for NGB and GB phosphenes) and essay responses (Figure S12aii,bii, Supporting Information; Table S1, Supporting Information) of human subjects. This suggests that, indeed, participants relied primarily on facial contour lines (i.e., face shapes), followed by hair or eyes, without consciously noticing their fixation points around the left side of the forehead, possibly formed unconsciously due to hemispheric lateralization.^[^ ^95^, ^96^ ^]^ This is also consistent with the claim from past research that humans tend to recognize phosphene patterns through holistic processing more than part‐based processing due to discarded details after down‐sampling.^[^ ^24^ ^]^

## 3 Discussion

In the present study, we have demonstrated the potential of utilizing machine learning (ML) models to estimate human recognition behaviors in response to low‐resolution phosphene images, with the aim of streamlining the laborious processes involved in human psychophysical tests for artificial vision research. While our findings from the comparisons between the human and ML recognition performances of faces rendered with two of the basic phosphene forms (i.e., NGB and GB) may serve as foundational benchmarks for future studies, several limitations must be addressed to enhance the translational implications.

Firstly, future investigations need to consider the intricate nature of phosphenes elicited during clinical trials. The perceptual manifestation of artificial can vary significantly depending on factors such as the type of stimulation (e.g., optogenetic^[^ ^52^, ^97^ ^]^ or electric^[^ ^31^, ^32^, ^34^, ^45^, ^46^, ^47^, ^54^, ^59^, ^63^, ^65^, ^67^, ^98^, ^99^ ^]^ stimulation) and the targeted visual centers (e.g., retina^[^ ^31^, ^32^, ^33^, ^34^ ^]^ or visual cortex^[^ ^40^, ^41^, ^42^ ^]^). Furthermore, phosphenes elicited by single‐electrode stimulation exhibit notable differences across subjects as well as depending on stimulus parameters.^[^ ^54^, ^55^, ^56^, ^57^, ^58^, ^59^, ^60^ ^]^ Therefore, although we believe we have established a foundation for the future replacement of human psychophysical tests for artificial vision, it is imperative to explore more realistic shapes of phosphenes, such as elongated streaks and blobs/wedges^[^ ^57^, ^68^ ^]^ using our methodology. Future work could explore finding or developing a model that aligns with human perception on complex phosphenes using *pulse2percept* simulation framework,^[^ ^100^ ^]^ which can better represent current real‐world settings. The collection of these irregularly shaped phosphenes may present substantial differences from those examined in the present study. Nonetheless, due to remarkable variability^[^ ^57^, ^68^ ^]^ (i.e., lack of consistency across subjects) and the unpredictable nature of actual phosphenes, we focused on testing NGB and GB phosphenes to showcase the feasibility of our approach. Our results suggest that psychophysical studies involving phosphenes in irregular shapes could be conducted using ML models as alternatives to human subjects. Additionally, exploring phosphenes in different colors may enhance the realism of our approach, as previous retinal prosthetic research reported color perceptions.^[^ ^101^, ^102^, ^103^, ^104^ ^]^ Color perception appears beneficial for object identification during natural viewing,^[^ ^105^, ^106^, ^107^ ^]^ and even more helpful for pixelized artificial vision because color can facilitate discrimination of subtle differences in visual features.^[^ ^101^ ^]^ Introducing color perception is likely to increase recognition accuracies in both humans and ML models as chromatic information provides more visual cues for object recognition.

Secondly, our study has focused exclusively on evaluating facial recognition accuracies of ML models using phosphene images without any background. However, the inclusion of natural or complex backgrounds may affect the performance of ML models, similar to the observed decrease in ML performance for high‐resolution images with natural (heterogeneous) backgrounds compared to those with uniform (homogeneous) backgrounds.^[^ ^1^ ^]^ Additionally, introducing natural backgrounds to our low‐resolution phosphene facial images may increase performance variability across subjects^[^ ^1^ ^]^ Moreover, to be useful for blind individuals in their daily lives, visual prostheses are expected to offer sophisticated artificial vision for diverse types of visual stimuli beyond faces. In addition to the facial recognition task, future studies could explore whether ML models can read sentences or letters,^[^ ^25^, ^72^ ^]^ recognize objects,^[^ ^22^, ^58^ ^]^ and identify rooms^[^ ^22^, ^108^ ^]^ similar to humans with phosphenized images represented in low‐resolution, corresponding to various stimulation methodologies.

Thirdly, our study has primarily focused on recognizing “*static*” faces using ML models as a potential replacement for humans in artificial vision research. However, given the “*dynamic*” nature of natural vision, the crucial next step for visual prosthetic researchers and developers would be to assess whether ML models can navigate in animated environments with object detection and facial recognition skills in natural scenes^[^ ^22^, ^58^ ^]^ To render psychophysical testing obsolete, it is essential to apply the proposed paradigm with ubiquitous objects commonly found in daily living environments. Additionally, to identify optimal image processing algorithms for simulating dynamic indoor/outdoor scenes in low resolution, head‐mounted displays (HMDs) could be utilized to superimpose artificial vision on the human subjects’ views while performing recognition and/or detection tasks, such as locating a chair among other furniture items in a dining room. For ML models, dynamic neural networks capable of processing video data by computing optimal parameter values across spatial and temporal dimensions could be constructed.^[^ ^109^ ^]^ Also, slow reading has been identified as a performance hurdle in clinical trials of retinal prostheses,^[^ ^39^, ^110^ ^]^ emphasizing the need for a fair estimation of reading speed in simulated artificial vision. Previous studies have computed the identification index defined as (recognition accuracy – chance probability) / correct response time.^[^ ^18^, ^21^, ^23^, ^25^, ^70^, ^71^ ^]^ However, in the present study, we analyzed the accuracies of facial recognition without normalizing it by the correct response time. This was because the ML models responded much faster than the human subjects, making a direct comparison unfair since the models did not exhibit any difference in correct response time between the initial and last test sets.

Our findings underscore the necessity of developing more effective ML models to accurately replicate human performances with unfamiliar low‐resolution artificial vision images. While Inception‐V3,^[^ ^2^ ^]^ Zeiler and Fergus^[^ ^1^, ^111^ ^]^ have been identified as the best‐performing DL models in terms of similarity with human behaviors for recognizing distorted objects in high‐resolution with synthetic background images, we found that the shallowest CNN (M05), or the Best Model, performed the best in terms of recognizing faces in low‐resolution phosphene images. Although the Best Model aligned well with human behavior data (Figure 2c), it is crucial to consider not only the accuracy but also the human consistency across differing facial classes, which was lacking in the models (as highlighted with red and blue arrows in Figure 4 and Figure S4, Supporting Information), in constructing view‐invariant *human‐like* models. Also, further refinement and application of improved ML models are required to address the performance gap found between recognition performances of optogenetically and electrically stimulated phosphenes, which was lacking in the humans (e.g., NGB vs GB; *compare* blue bars in Figure 3ai vs Figure S3ai, Supporting Information, and *compare* red bars in and Figure 3aii vs Figure S3aii, Supporting Information). To achieve these goals, extensive experiments are needed to identify artificial neurons tuned/selective to essential abilities (e.g., facial recognition, number discrimination),^[^ ^81^, ^82^, ^112^ ^]^ which can then be used to improve model performance. For instance, efforts could be made to identify face‐selective artificial neurons in our trained deep neural networks, which correspond to real neurons in the fusiform face area (FFA) located in the inferior temporal (IT) cortex, and leverage them to enhance the performance of the Best Model.^[^ ^82^ ^]^ Also, it has been demonstrated that noise‐trained DL models can perform comparably to human performances,^[^ ^10^ ^]^ suggesting the performance gaps between subjects and models can be further reduced by training models with phosphene images to closely match human performance, particularly for low‐resolution phosphenes (Figure 2c and Figure S2c, Supporting Information for NGB and GB cases). However, fine‐tuning DL models with facial contour images did not improve performance (Please note that this fine‐tuned DL does not refer to M13).

In our study, we compared the behaviors of human subjects with ML models that were not fine‐tuned. While fine‐tuned models are commonly used to enhance accuracy when testing with distorted or noise‐added data,^[^ ^1^, ^2^, ^9^, ^10^, ^11^ ^]^ their underlying mechanism involves feeding data with a distribution closer to test images into the trained model, potentially introducing bias by favoring the models over humans who lack experience in interpreting artificial vision.^[^ ^113^ ^]^ Therefore, we opted to compare nonfine‐tuned models with first‐time participants with no prior experience in interpreting artificial vision. Since we recruited normally sighted human participants, training the models with original high‐resolution images to mimic human adults with innate facial recognition abilities was deemed appropriate. However, when estimating the performances of implant recipients who still retain natural peripheral vision,^[^ ^53^ ^]^ fine‐tuning the models may be necessary since the functional peripheral vision can be well integrated with new artificial vision at the center of the visual field.^[^ ^114^ ^]^

Interestingly, humans tend to perceive similarly across the two different types of stimuli we tested (i.e., NGB vs GB; *note* the minimal differences between red bars in Figure 2c and Figure S2c, Supporting Information), consistent with the Weber‐Fechner law.^[^ ^115^ ^]^ Although we demonstrated that the performances of the Best Model (i.e., M05) with 16 facial classes were comparable to those of humans with 4 multiple choices in recognizing faces from NGB phosphene images, it remains a challenge to find the most optimal model that is generalizable to any kind of artificial vision (e.g., GB version in this paper or noncircle phosphenes^[^ ^20^, ^57^ ^]^). Although M05 with 16 facial classes could follow the trend of human performances across PIQ levels for both NGB and GB phosphene images, the performance gaps were evidently noticeable, especially in low resolutions up to 32 × 32 in recognizing faces from GB phosphene images (*compare* blue bars in Figure 2c and Figure S2c, Supporting Information). Therefore, it seems crucial to develop a reliable ML model that not only mirrors the human recognition trend along various PIQ levels for one type of artificial vision but also performs well for any type of artificial vision simulating diverse stimulation types (e.g., NGB and GB for optogenetic and electric approaches).

The social cost of blindness is estimated to be $20 billion globally,^[^ ^116^ ^]^ yet the best quality of artificial vision provided by visual prostheses remains significantly below that of normal vision despite various prosthetic approaches.^[^ ^34^, ^44^, ^45^, ^46^, ^47^, ^48^, ^49^, ^52^ ^]^ The main challenge stems from attempting to recreate an incredibly sophisticated visual system of primates capable of generating exquisite and dynamic visual perceptions in real‐time.^[^ ^117^ ^]^ To achieve practically useful artificial vision enabling complicated visual tasks, such as independent navigation and facial recognition, visual prosthetic devices may need to faithfully replicate various neurophysiological aspects of natural vision at the cellular level, including unique spiking activities in each visual neuron,^[^ ^118^, ^119^, ^120^ ^]^ selective activation of task‐specific neurons,^[^ ^121^ ^]^ and the processing of vast amounts of neural information.^[^ ^122^ ^]^

This study contributes to prosthetic advancement by focusing on the system level, demonstrating the feasibility of utilizing ML models to complement conventional human experiments of artificial vision. Traditional experiments with human subjects often entail extensive testing of diverse stimulation design parameters such as pixel count and grayscale levels,^[^ ^25^, ^26^, ^58^ ^]^ necessitating repeated experiments to obtain reliable results. Instead, our novel experimental paradigm employing ML models offers a promising approach to not only reduce labor costs associated with psychophysical tests but also accelerate the overall development of prosthetic systems. We demonstrated the potential of ML approaches to enable efficient estimation of the recognition abilities of prospective prosthetic users using our proposed prediction approach. This methodology could serve as a baseline, paving the way for the broader adoption of ML and AI in the field of visual prosthetics. Perhaps, gaining a comprehensive understanding of how deep neural network models decipher low‐resolution phosphene images may offer valuable insights into brain cognitive function related to low‐resolution visual stimuli,^[^ ^81^ ^]^ and vice versa. Such insights could further enhance our understanding of artificial vision and contribute to their continued improvement and refinement.

## 4 Experimental Section

### Generation of facial images with phosphene arrays

The K‐Face database (https://www.aihub.or.kr/aihubdata/data/view.do?currMenu=115&topMenu=100&aihubDataSe=realm&dataSetSn=83=115&topMenu=100&aihubDataSe=realm&dataSetSn=83) was used,^[^ ^123^ ^]^ compiled from 2017 to 2019 by the Artificial Intelligence and Robotics Institute at the Korea Institute of Science and Technology (KIST). This dataset comprises facial images of 400 South Koreans (210 males and 190 females; further demographics are summarized in Figure S7a, Supporting Information). All individuals in the dataset were new to the human subjects who participated in our study, ensuring an examination of recognition ability for unfamiliar faces without any memory effect.^[^ ^9^, ^18^ ^]^ Each person had 27 image variations, such as different lighting conditions, viewpoint angles, accessories, and facial expressions, resulting in a total of 10 800 images. From the 400 faces given, 137 faces were randomly selected and variations that were barely recognizable were removed due to dark lighting or excessive accessories, such as sunglasses, from the original high‐resolution images (Figure S7b, Supporting Information), resulting in 4872 photos per facial class. Then, squares surrounding faces from the images (Figure S7c, Supporting Information) were cropped. To focus exclusively on the faces themselves without any contextual or background elements of the images,^[^ ^81^ ^]^ all backgrounds were removed using facial masks generated by applying our K‐Face dataset on a trained U‐Net model^[^ ^17^ ^]^ (Figure S7d, Supporting Information). The U‐Net model comprises a pretrained VGG16 encoder^[^ ^15^ ^]^ and was re‐trained using the CelebA‐HQ dataset^[^ ^124^ ^]^ (900 celebrities for training, 100 celebrities for validation). Hair was retained in the images as it is known to be a helpful feature for recognizing faces.^[^ ^21^ ^]^

For training the ML models, original high‐resolution images with dimensions of 128 × 128 pixels and 256 grayscale levels (ranging from 0 to 255) were utilized. Low‐resolution phosphene images were used to test the ML models. Initially, from the pool of 4872 image variations per facial class, several key parameters were manually selected, including three viewpoint angles (i.e., –45, 0, and +45 degrees) and three facial expressions (i.e., neutral, joy, and anger), resulting in 3600 test images per facial class. Notably, for the human experiment, a subset of these parameters, comprising neutral, joy, and anger expressions at 0 degrees and additional neutral expressions at −45 and +45 degrees, was chosen, resulting in 720 test images for human subjects. Subsequently, all test images were converted to grayscale since color perception is not possible, in general, with state‐of‐the‐art artificial vision technologies.^[^ ^44^, ^45^, ^46^ ^]^ Furthermore, the contrast of the images was enhanced by spreading pixel values into a uniform distribution^[^ ^23^ ^]^ (referred to as “Histogram‐equalized” in Figure S7e, Supporting Information). Following a methodology similar to previous studies,^[^ ^18^, ^21^, ^22^, ^23^, ^25^, ^58^, ^70^, ^71^, ^72^ ^]^ the numbers of pixels were varied (16 × 16, 24 × 24, 32 × 32, 64 × 64, and 128 × 128) and along with grayscale (GS) levels (2, 4, 6, 8, and 16), resulting in a total of 25 “PIQ levels” (Figure S7fi, Supporting Information). The selection of 1616 pixels as the minimum pixel count was based on previous findings suggesting that this dimension is challenging for recognition even for individuals with normal vision.^[^ ^11^ ^]^

To investigate the impact of different stimulation approaches on facial recognition accuracy, two distinct configurations of stimulations were simulated – non‐Gaussian blurred (NGB; represented as square bitmap) phosphenes (Figure S7fi, Supporting Information) and Gaussian blurred (GB) circular discrete phosphenes (Figure S7fii, Supporting Information), both applied on a square lattice map.^[^ ^20^ ^]^ The former simulates the optogenetic stimulation, where light pulses are projected onto the retina,^[^ ^52^, ^97^ ^]^ assuming that one phosphene corresponds to one uniformly contrasted pixel. In the latter configuration, each phosphene exhibits maximum brightness at the center and gradually diminishes in brightness toward the periphery of a given pixel, forming a circular shape^[^ ^31^, ^32^, ^34^, ^45^, ^46^, ^47^, ^54^, ^59^, ^63^, ^65^, ^67^, ^98^, ^99^ ^]^ For the downsampling process, the number of pixels were reduced using nearest‐neighbor interpolation. To ensure uniform input sizes for images with varying numbers of pixels, nearest‐neighbor interpolation was employed to upscale the images for the NGB version and convolved the images with kernels for the GB version (Figure S7gi‐giii, Supporting Information; note the larger kernel size in Figure S7gi than Figure S7gii,giii, Supporting Information).

### Machine Learning Models

Various types of ML models were tested, including PIXEL (M01 and M02), principal component analysis^[^ ^88^ ^]^ (PCA; M03 and M04), convolutional neural network (CNN; M05 and M06), AlexNet^[^ ^15^ ^]^ (M07 and M08), visual geometry group (VGG;^[^ ^17^ ^]^ M09 and M10) and residual neural network (ResNet;^[^ ^89^ ^]^ M11 and M12) (Figure S1a, Supporting Information). CNN‐based models over Vision Transformers (ViTs) were prioritized due to the inherent advantages of CNNs in capturing local patterns, such as edges, through strong inductive biases. This characteristic enables CNNs to more effectively extract features from low‐resolution phosphene images, where fine details are limited.^[^ ^11^, ^125^, ^126^, ^127^ ^]^ In contrast, ViTs exhibit challenges in processing low‐resolution inputs because partitioning images into patches—proportional to the image resolution—restricts the effective encoding of semantic features within the Multi‐Head Self‐Attention mechanism.^[^ ^128^ ^]^ PIXEL serves as a baseline ML model that does not extract any features.^[^ ^1^, ^2^ ^]^ For PCA, it was confirmed that 128 principal components must explain at least 90% of the original data in a 128 × 128 dimension. In the case of DL models, the architectures used in the original papers were modified^[^ ^15^, ^17^, ^89^ ^]^ (Figure S8, Supporting Information). Since end‐to‐end training is more efficient in finding optimized parameters for each layer compared to greedy layer‐wise training,^[^ ^11^ ^]^ implementing an end‐to‐end training approach for the DL models was focused. Most models comprised a convolution (Conv) layer, followed by either a max‐pooling (MaxPool) or a global average‐pooling (GAvgPool) layer, along with a rectified linear unit (ReLU) activation function and a batch‐normalization (BN) layer for adjusting the output values. The architecture of ResNet was structured into residual blocks concatenated with two Conv layers and one Conv identity layer. While the ML models utilized different feature extraction encoders, they all included the same single‐layer neural network of classifiers with either 16, 4, or 2 artificial neurons, depending on the number of facial classes. Additionally, M13 shared the same CNN architecture as M05/M06 but was trained in different procedures (*see* the next section for details).

### ML Model Training Procedures

For each model architecture (M01‐M13), one thousand random images per facial class were used to train each model instance, creating five to ten different model instances. The dataset was randomly divided into training and validation sets in an 85:15 ratio. 14 test subsets were generated from the same K‐Face dataset (Figure S9ai, Supporting Information), each with a distinct demographic distribution, including gender and age (Figure S9aii, Supporting Information). For instance, certain sets exclusively included females aged between their 20s and 30s (e.g., Sets 2 and 9), while other sets included males aged between their 20s and 50s (e.g., Sets 7 and 14). Set #1 was designated as the primary test dataset due to consistent overall facial recognition accuracy of the Best Model across different sets (i.e., no statistical significance was observed across different sets; Figures S9,bii, Supporting Information, for the NGB and the GB versions, respectively), and there was no observed gender or age effect (Figures S9ci,cii, Supporting Information, for the NGB and the GB versions, respectively).

During the training process, a single GPU (GeForce RTX 3070, NVIDIA, Santa Clara, CA) was employed, and each ML model was optimized using two different types of loss functions – cross‐entropy loss (CEL)^[^ ^129^ ^]^ and multimargin loss (MML).^[^ ^130^ ^]^ The former maximizes the log‐likelihood of estimated probability,^[^ ^129^ ^]^ while the latter maximizes the margins of hyperplanes that separate training data by different facial classes.^[^ ^130^ ^]^ The total number of epochs was set to 30, but the training procedure stopped if the current validation loss did not decrease by 0% for seven to ten consecutive epochs compared to the lowest validation loss observed until that epoch. The batch size was set to 16, and the Adam optimizer with an initial learning rate of 110^−4^ and a weight decay of 110^−4^ was used. The learning rate was reduced with a factor of 110^−2^ when the current validation accuracy did not exceed the highest validation accuracy observed until that epoch within five epochs. The minimum learning rate was set to 110^−8^. Only the fine‐tuned DL model, M13, was trained with 500 high‐resolution and 500 NGB images at the highest PIQ level (128 × 128 with 16 GS). Specifically, half of the 1000 training images were downgraded to NGB versions, while the other half remained unchanged during training.^[^ ^9^ ^]^ The Pytorch framework from Python was utilized to train all the ML models.

### Human Participants

36 South Korean subjects were recruited (10 males and 8 females indicated by H01‐H18 for the NGB version and 6 males and 12 females indicated by H19‐H36 for the GB version; demographics are shown in Figure S10, Supporting Information) over four different days. The total tests took about two hours on average per participant, ranging from ≈1 h 42 min to 2 h 30 min (≈2.09 h on average with 10.40 min of standard deviation; a sample video is included as Movie S1, Supporting Information). The human psychophysical experiment was approved by the Institutional Review Board (IRB) of the Korea Institute of Science and Technology (KIST). All participants were provided information about the experimental procedures and consented before starting their tests. They also underwent a visual acuity test by standing four meters away from an Early Treatment Diabetic Retinopathy Study (ETDRS) Chart R (62 × 65 cm^2^)^[^ ^131^ ^]^ to ensure their corrected vision in the logMAR scale was less than or equal to 0.3, indicating that they did not fall within the range of low vision according to the World Health Organization.^[^ ^132^ ^]^ Upon completion of the whole set of psychophysical tests, participants received monetary compensation (15000 Korean Won, equivalent to ∼$11.39 as of March 10, 2024) per hour as a participation reward.

### Human Behavioral Protocol

Psychophysical tests were conducted using the online website on laptops (MSI GP63 Leopard; Intel Core i7‐8750H). The experiments were carried out in a carefully controlled lab environment: cardboard compartments were placed on the experiment tables to prevent participants from being distracted by other computer screens. Subjects were instructed to maintain fixed head postures to ensure a consistent field of view of 7 degrees (*d* = 60 cm, *w* = *h* = 7.33 cm) throughout the experiment.^[^ ^25^, ^133^ ^]^ To maintain their concentration during the tests, participants were given optional five‐minute break periods between each set, which consisted of 80 questions. The test was divided into three main sessions: 1) Pretest, 2) Main test, and 3) Post‐test (Figure S11ai‐aiii; a sample video is attached as Movie S1, Supporting Information). The Pretest session (Figure S11ai, Supporting Information) aimed to familiarize human subjects with the experimental procedures, although this session was not necessary for machines since they had the inherent computational power to perform tasks immediately without familiarizing themselves with the environments.^[^ ^134^ ^]^ The tiny‐ImageNet database distributed by Princeton University and Stanford University (https://www.image‐net.org/index.php)^[^ ^135^ ^]^ for the Pretest stage was used, which was terminated after participants provided five correct answers, not necessarily consecutively.

The Main test consisted of 720 trials (9 PIQ levels, 16 facial classes per PIQ level, and 5 facial conditions such as different viewpoint angles and facial expressions for each facial class; Figure S11aii, Supporting Information). Consecutive test images at different PIQ levels were presented (i.e., resolution and grayscale) to minimize the learning effect of memorizing faces^[^ ^18^ ^]^ Additionally, each participant never encountered the same test image (i.e., a given face expressed in an identical PIQ condition) more than once, and each face was shown only 1–3 times (i.e., but in different PIQ conditions).^[^ ^2^ ^]^ For each question in the Main test, a low‐resolution phosphene image was displayed for three seconds. Although research suggests that the human brain can decode object category information in 100 ms post‐stimulus,^[^ ^136^ ^]^ a longer viewing time was allowed, considering the challenges posed by low‐resolution phosphene images^[^ ^39^, ^58^ ^]^ Following the presentation of the image, a one‐second blank white screen was shown to introduce a delay in stimulus onset asynchrony (i.e., the time period between the first stimulus and the next stimulus).^[^ ^137^ ^]^ This delay screen is essential for participants to fixate on a new visual stimulus appearing on the screen after the delay, as the visual system cannot fully process the previous visual stimulus without a break between stimuli.^[^ ^1^ ^]^

During the Main test, participants were presented with a four‐way forced multiple‐choice (MC) screen containing original high‐resolution for up to 5 seconds. If participants answered more quickly than 5 seconds, they could proceed to the next screen. Six different sets of MC questions (i.e., MC1‐MC6) were prepared for the 36 human subjects, with each subject randomly assigned to one of the MC question sets. Consequently, six human groups (G01‐G06) were formed, with each group comprising six individuals who had seen the same MC screens. Specifically, the first three human groups, G01 (H01‐H06), G02 (H07‐H12), and G03 (H13‐H18), were tested with the NGB phosphenes, while the remaining three human groups, G04 (H19‐H24), G05 (H25‐H30), and G06 (H31‐H36), were tested with the GB phosphenes. For example, the subjects in G01 were presented with MC1 screens, distinct from MC2 and MC3 screens.

After the MC screens, participants were prompted with a binary confidence level screen (high/low) that appeared with a time limit of three seconds. This screen aimed to capture the second most likely answer participants would select if they clicked the “low” confidence level. A binary confidence level setting was opted due to potential variability in participants’ confidence criteria if more than two options were provided. For instance, using a scale of 1 (i.e., 0% confident) to 5 (i.e., 100% confident)^[^ ^58^ ^]^ might result in inconsistent responses, as individuals could interpret confidence levels differently. In other words, if two people are very confident, one might choose 5, and the other might choose 4, leading to noisy and unreliable confidence level results.

While the facial recognition performances of 36 human subjects were automatically recorded during the Main test stage (Figure S11aii, Supporting Information), their responses to the six fixed multiple‐choice questions were additionally collected to assess their subjective preferences with low‐resolution phosphene images (Figure S11aiii, Supporting Information). For these questions in the Post‐test stage, two facial classes in 4 GS were presented in the three resolution levels of 16 × 16, 32 × 32, and 64 × 64. For each multiple‐choice question, there was no time limit, unlike the Pretest stage (Figure S11ai, Supporting Information) and the Main test stage (Figure S11aii, Supporting Information). Participants were asked whether they could recognize a human face from the given low‐resolution phosphene image and which facial feature they mostly focused on when trying to recognize the face. They were instructed to choose only one option out of eight features: facial lines, eyes, hair, nose, mouth, ears, eyebrows, and nonfacial features. It was confirmed that it took more than 1.4 seconds per trial throughout test datasets for all resolution levels (Figures S11ci,cii, Supporting Information, for NGB and GB phosphenes, respectively).

### Word Cloud Visualizations of the Subjective Opinions of Human Subjects

Subjects were invited to provide their responses to short‐answer questions during the break times or after completing the entire test. Subjective opinions from 35 out of 36 human subjects (with one subject not responding to our inquiries) were obtained regarding the difficulty level of the psychophysical test and the facial features they primarily focused on while viewing phosphene images. These responses, originally in Korean, were translated into English using DeepL (https://www.deepl.com/en/translator) and are included in the Supplementary Information (Table S1, Supporting Information). Word clouds were generated using the word cloud package in Python^[^ ^138^ ^]^ (Figures S12a,b, Supporting Information, for NGB and GB phosphenes, respectively), with a maximum of 50 words displayed in the clouds. Some words were excluded from the word clouds, including “very,” “quite,” “the,” “it,” “even,” “think,” “etc.,” “will,” “given,” “whenever,” “overall,” “felt,” “much,” “clearly,” “sure,” “several,” and “see.” The full list of the excluded words can be found in our code at the following link: https://github.com/namin‐an/ArtificialVision/blob/main/Visualization/Wordclouds.ipynb

### Prediction of Human Recognition Accuracy Using ML Models

The linear and nonlinear relationships were established between facial recognition accuracies of ML models and human subjects in tested PIQ levels to estimate human recognition accuracies for nontested PIQ levels, such as 24 × 24 and 32 × 32 with 6 GS, from the accuracies of ML models for those PIQ levels (i.e., 24 × 24 and 32 × 32 with 6 GS). For the linear approximation, a regression line was fitted to plot the accuracies of human subjects (on the *y*‐axis) against those of ML models (on the *x*‐axis) for the PIQ levels tested in both humans and machines (Figures 3bi and Figure S3bi, Supporting Information, for NGB/GB cases). Using the estimated linear curve, human performance estimations were derived from the results of ML models in the PIQ levels tested in machines.

In the case of the nonlinear approach, input vectors were constructed by concatenating the accuracy of the Best Model instance per facial condition and its correlation with a human subject. These vectors were then fed into a CNN with a single hidden layer, referred to as the shallow ML model (Figures 3bii and Figure S3bii, Supporting Information for NGB/GB cases). Five facial conditions were used (three viewpoint angles of −45, 0, and +45 degrees, and two facial expressions – joy and neutral). In the hidden layer, three or four artificial neurons were allocated for the NGB or GB cases, respectively. The output of the shallow ML model was a 1D vector comprising accuracy per facial condition for the corresponding human subject. The training of the shallow ML model involved 1620 trials, encompassing combinations from 18 human subjects, 10 model instances, and 9 PIQ levels, depending on the scale of human and ML model experiments. Unlike the main ML training procedure, Keras in Python was implemented to train the shallow ML model. The model was compiled using the mean squared error as the loss function and the Adam optimizer. During the model fitting, the batch size was 16, the epoch was set to 3k, and the train‐validation split ratio was 9:1.

### Saliency Maps for ML Models and Humans

Grad‐CAM was employed to interpret DL models by highlighting the significant features of human faces in test images that the models focused on when making decisions.^[^ ^94^ ^]^ Additionally, eye‐tracking sensors (Tobii Pro Fusion 120Hz, Tobii Technology, Danderyd Municipality, Sweden) were utilized to monitor the eye movements of five human subjects for each stimulation type (participants H02, H05, H07, H10, and H13 for the NGB version; participants H21, H24, H28, H31, and H34 for the GB version). It is important to note that all participants with eye trackers completed different questions (two participants from G01/G04, another two participants from G02/G05, and one participant from G03/G06 for the NGB/GB versions, respectively; demographics are shown in Figure S10, Supporting Information). The average relative duration of fixation for each test image was visualized using heatmaps (Figure S11b, Supporting Information). Warmer colors in the heatmap indicate longer fixation durations (where fixation was flagged if the velocity of eye motion, measured in visual degrees, was below a default threshold of 30 degrees per second set in the Tobii Pro Lab software program).^[^ ^139^ ^]^

### Metrics for Evaluating Facial Recognition Accuracy and Performance Similarity for ML Models and Human Subjects

The Top‐1 facial recognition accuracies of both ML models and human subjects were defined as the ratio of correct responses expressed as a percentage. Additionally, the Top‐2 accuracy was computed, where for ML models, it considered the two highest probabilities, and for human subjects, it allowed a second chance if they were not confident in their first choice (Figure S11ai,aii, Supporting Information). Facial discriminability levels were determined by averaging the Top‐1 facial recognition accuracy (%) across specific conditions (e.g., joyful face) within a facial class (e.g., F03) for each model or a human group, with red and blue plots corresponding to 0 and 100, respectively (Figure 4a and Figure S4a, Supporting Information).

Response time was measured only for correct answers provided by human participants, resulting in correct response time.^[^ ^18^, ^21^, ^23^, ^25^, ^70^, ^71^, ^72^ ^]^ To quantify the behavioral similarity between the Best Models with two different numbers of facial classes (Figure 2b and Figure S2b, Supporting Information), as well as between human subjects and ML models (Figure 3bi and Figure S3bi, Supporting Information), Pearson's correlation coefficient (Pearson's *r*) was used. Unlike previous studies,^[^ ^1^, ^9^ ^]^ the level of distortions was not explicitly defined (variations) (i.e., how much an image is distorted with various parameters – rotation, eccentricity, etc.) since it was challenging to determine the superiority or inferiority of certain PIQ levels (e.g., 32 × 32, 4 GS) compared to others (e.g., 64 × 64, 2 GS) when both numbers of pixels and grayscale levels were simultaneously altered.^[^ ^18^, ^25^ ^]^

### Statistical Analysis

1. **Preprocessing of data**: To compare machine learning model data to human experimental data, output logits was normalized to calculate Top‐1 and Top‐2 facial recognition accuracies for the models. For humans, Top‐1 accuracy was determined by the first choice among four presented facial class options, and Top‐2 accuracy was calculated based on the second choice from the remaining three options. To ensure the reliability of the data, each model was independently tested 10 times, while 18 different human subjects were tested with the same set of questions for each phosphene type. Data points deviating by more than 2 standard deviations from the mean across independent model runs and human subjects were excluded.
2. **Data presentation**: All the numerical data are presented with mean ± standard deviation.
3. **Sample size for each statistical analysis**: For machine learning models, 5 resolutions, 5 grayscale levels, 16 facial classes, and 9 facial attributes were tested, resulting in a total sample size of 3600 per model. For human participants, due to time and resource constraints, 3 resolutions, 3 grayscale levels, 16 facial classes, and 5 facial attributes were tested, resulting in a total sample size of 720 per participant.
4. **Statistical methods**: The one‐sided Wilcoxon Rank Sum Test, also known as the Mann‐Whitney U Test, was employed to assess the degree of similarity between two independent distributions of performances (i.e., facial recognition accuracies) in humans and ML models. The nonparametric test was used since the number of independent runs (e.g., ten model instances and eighteen human subjects) was considered insufficient (less than 30 data points) to follow the Central Limit Theorem. Our statistical test on Figure 2c can be specifically found at https://github.com/namin‐an/ArtificialVision/blob/main/Visualization/Parallel.ipynb.
5. **Software used for statistical analysis**: All statistical analyses in this paper were conducted using the Python package SciPy.

### Human Subject Data and Code Availability

All the data from the human psychophysical experiments, along with the Python codes for image preprocessing and model analyses, have been uploaded to https://github.com/namin‐an/ArtificialVision. However, the scanned copies of the subjective opinions of 35 human subjects (1 out of 36 subjects left his/her answer sheet blank) were excluded from the abovementioned link. This exclusion is due to the following reasons: 1) the responses were written in Korean (English translations are shown in Table S1, Supporting Information), and 2) handwriting could be considered personal data. Those interested in accessing these responses may be provided upon reasonable requests via e‐mail to the corresponding author, provided that an appropriate nondisclosure agreement is in place.

## Conflict of Interest

The authors declare no conflict of interest.

## Supporting information



Supporting Information

Supplemental Movie 1

## Data Availability

The data that support the findings of this study are available from the corresponding author upon reasonable request.
