# Supplementary material for: Machine Learning Techniques for Simulating Human Psychophysical Testing of Low‐Resolution Phosphene Face Images in Artificial Vision
Source: Adv Sci (Weinh). 2025 Feb 22;12(15):2405789. doi: 10.1002/advs.202405789 (PMC12005743; doi:10.1002/advs.202405789)
Supplement: Supplementary file 1 — Supporting Information [file ADVS-12-2405789-s001.docx]

Supporting Information

Machine Learning Techniques for Simulating Human Psychophysical Testing of Low-Resolution Phosphene Face Images in Artificial Vision

Na Min An, Hyeonhee Roh, Sein Kim, Jae Hun Kim, and Maesoon Im^*^

**Supplementary Figures**

**
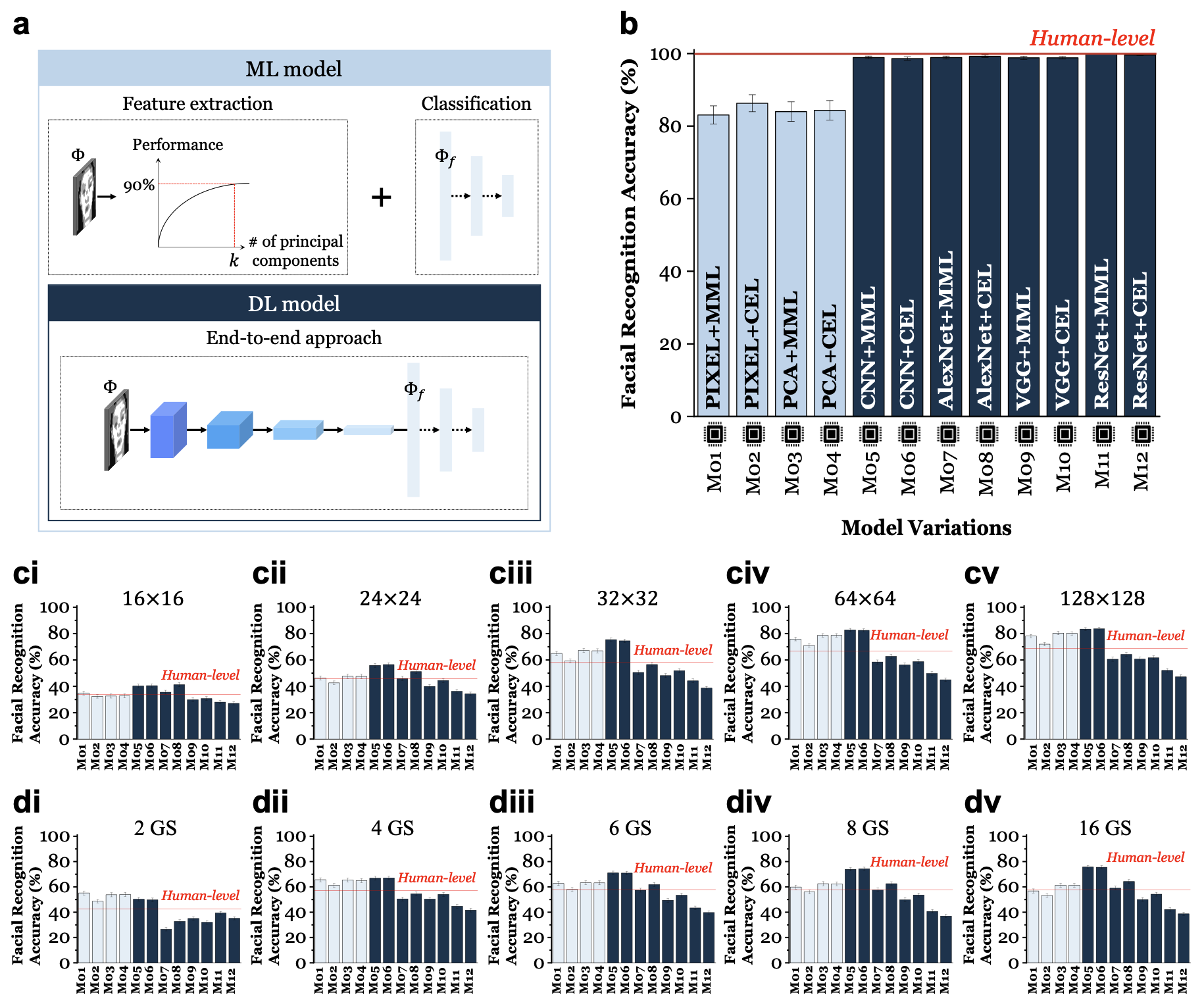
**

**Fig. S1. | Performance evaluation of machine learning (ML) models using high-resolution test images. (a)** The present study used 12 different ML models (M01-M12), categorized into conventional ML models (M01-M04) and end-to-end deep learning (DL) models (M05-M12). The conventional ML models included PIXEL (M01/M02) and principal component analysis (PCA) (M03/M04); feature extraction and classification components were trained separately. The DL models encompassed convolutional neural network (CNN) (M05/M06), AlexNet (M07/M08), Visual Geometry Group (VGG) (M09/M10), and Residual Neural Network (ResNet) (M11/M12). Each ML model utilized either multi-margin loss (MML) or cross-entropy loss (CEL) as a loss function. **(b)** Conventional ML models (light blues) achieved over 80% accuracy in identifying the correct face out of 16 multiple-choice facial classes, while DL models (dark blues) achieved near-perfect facial recognition accuracy, comparable to humans (*n* = 1,000 for each model). It is important to note that original high-resolution test images were utilized here, maintaining the same data distribution as the training images. **(ci-cv)** **Figures 2ai-axii** were visualized as 2D versions by averaging over the number of pixels. **(di-dv)** **Figures 2ai-axii** were visualized as 2D versions by averaging over the number of grayscale levels.

**
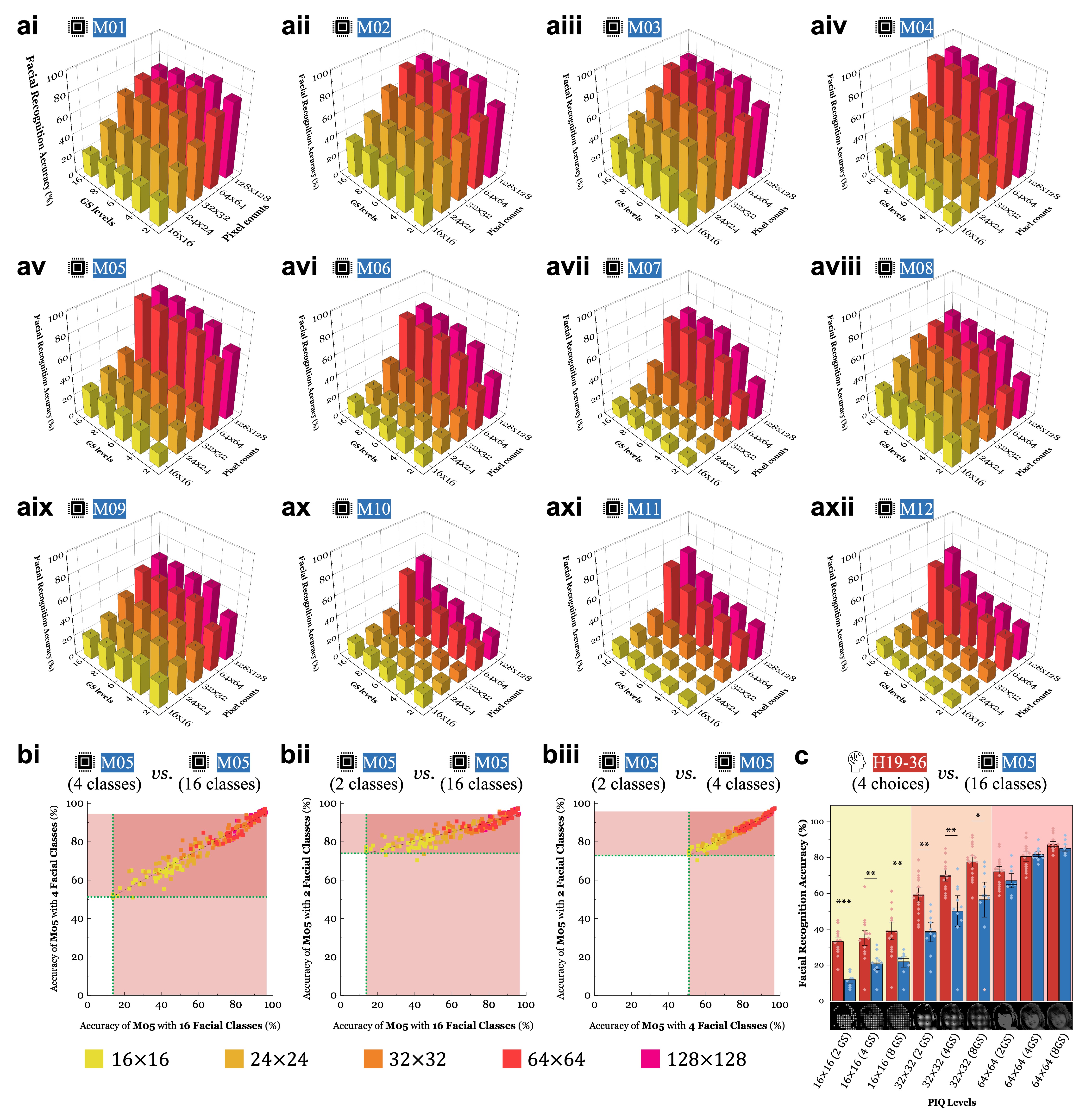
**

**Fig. S2. | For the phosphene images in Gaussian-blurred (GB) version, the facial recognition accuracy of machine learning (ML) models mirrors that of humans. (ai-axii)** Twelve ML models were tested to determine their ability to correctly recognize faces out of 16 facial classes across 5 resolutions and 5 grayscale levels (referred to as phosphene image quality; PIQ levels). For most models, resolution levels (*i.e.*, pixel counts) had a more significant impact on facial recognition accuracy compared to grayscale levels. Model M05, identified as the Best Model, consistently outperformed other models across nearly all PIQ levels, except for M03/M04 at the pixel counts of 16×16 and 32×32. Error bars in each panel represent the standard errors of 16 facial classes and 9 facial conditions. **(bi-biii)** The Best Model exhibited a larger range (band gap) of accuracy when tested with a relatively greater number of facial classes (*e.g.*, 16) compared to a small number of facial classes (*e.g.*, 2 or 4). Green dotted lines indicate the minimum recognition accuracy for the lowest-resolution images for each given number of facial classes. However, high linear correlations exist across 25 PIQ levels between M05s tested with two different numbers of facial classes as follows: **(bi)** 16 and 4 (*r* = 0.96, *n* = 225, *p* < 0.001), **(bii)** 16 and 2 (*r* = 0.90, *n* = 225, *p* < 0.001), and **(biii)** 4 and 2 (*r* = 0.97, *n* = 225, *p* < 0.001). Therefore, the performance order remained consistent even with modifications to the number of artificial neurons in the last layer. **(c)** The facial recognition accuracy of M05 with a facial class size of 16 closely matched human performance across 3 out of 9 (33%) PIQ levels. Each bar indicates the average and one standard deviation of 18 human subjects and 10 model instances (*i.e.*, independent runs) (^*^*p* $<$ 0.05, ^**^*p* $<$ 0.01, ^***^*p* $<$ 0.001, Wilcoxon rank sum test). Human brain icon was obtained from Flaticon.com.

**
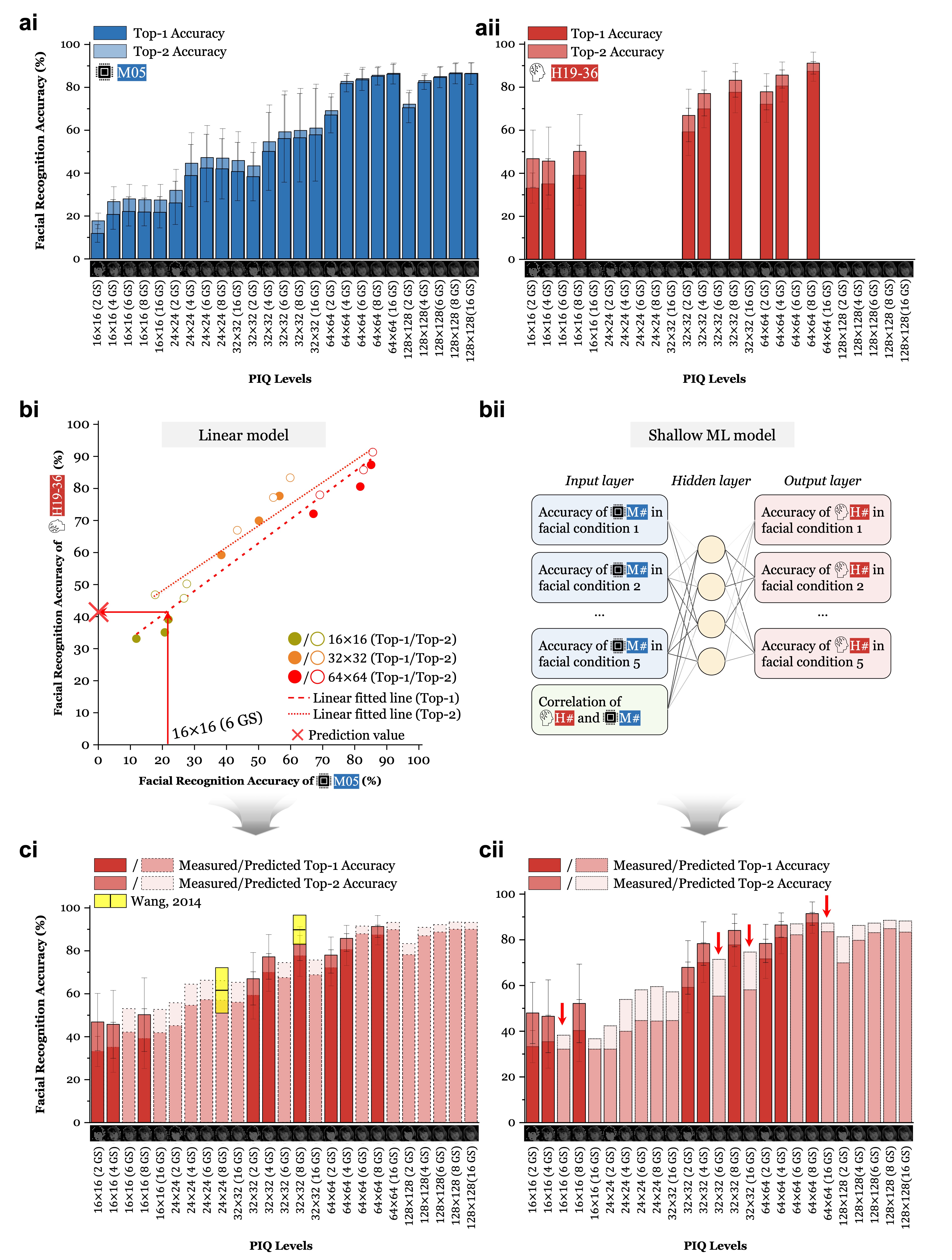
**

**Fig. S3. | Prediction of human performances at unmeasured phosphene image quality (PIQ) levels using the strong correlation between the recognition performances of humans and machine learning (ML) models for Gaussian-Blurred (GB) version phosphene face images.** Top-1 and Top-2 facial recognition accuracies of **(ai)** the Best Model (*i.e.*, M05) and **(aii)** human participants. Each bar represents the average and one standard deviation of 80 (16 facial classes × 5 facial conditions) and 144 (16 facial classes × 9 facial conditions) test images for the humans and the M05, respectively. The human subjects (*n* = 18) were tested for only 9 PIQ levels, while the Best Model instances (*n* = 10) were tested for all 25 PIQ levels. The Best Model accuracies were used to predict the remaining 16 PIQ levels in human psychophysical testing, totaling 2,880 test images (16 PIQ levels × 16 facial classes × 9 facial conditions). **(bi)** Given the comparable performance trends observed between the averages of 18 human subjects and 10 model instances across the nine tested PIQ levels for both Top-1 (*r* = 0.96, *p* < 0.001) and Top-2 accuracies (*r* = 0.96, *p* < 0.001), a linear regression was applied to the data. The resulting linear fitting curves are depicted by a dashed red line for Top-1 accuracy and a dotted red line for Top-2 accuracy, facilitating the estimation of human accuracy values at unmeasured PIQ levels (*e.g.*, 16×16 with 6 grayscale (GS) levels). **(bii)** Additionally, a non-linear model, specifically the shallow ML model, was utilized to characterize the relationship between the Best Model instances and the humans using the accuracy of each human subject/model instance and their correlation levels. Subsequently, face recognition accuracies of humans for the unmeasured PIQ levels were estimated using both linear and non-linear methodologies, respectively. **(ci)** For two PIQ levels, the predicted values of human performances generated by the linear model closely followed the trend of recognition accuracies observed in a previous study (Wang *et al*., *J. Neural Eng.*, 2014) ^[21]^. Yellow bars represent the mean (indicated by black lines in the middle) and standard deviations. **(cii)** The non-linear model produced several estimation values that were notably lower than neighboring measured values, indicating that the linear model might suffice for predicting performances unmeasured in psychophysical testing. Human brain icon was obtained from Flaticon.com.


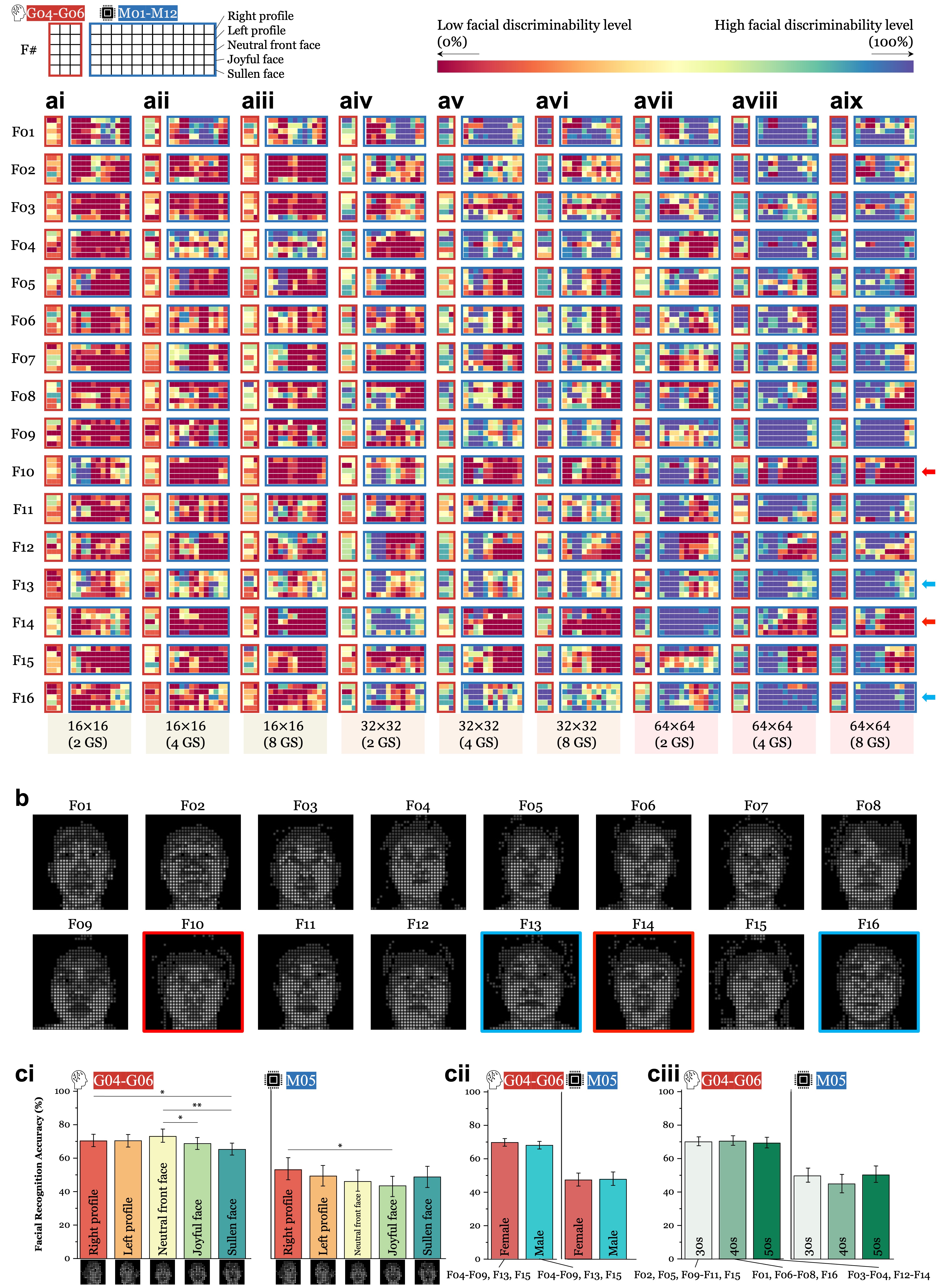


**Fig. S4. | Differences in image-level facial discriminability between humans and machine learning (ML) models for Gaussian-Blurred (GB) version phosphene images. (ai-aix)** Both human groups (G04-G06) and ML models (M01-M12) struggled to recognize most of the test images at the lower resolution (*i.e.*, 16×16), as evidenced by the prevalence of warm-colored bins. However, the human groups exhibited progressively better performance as the phosphene image quality (PIQ) level increased, with few or no red squares at the resolution level of 64×64. In contrast, the ML models still occasionally showed red squares at the higher resolutions (*i.e.*, 64×64). From the PIQ level of 32×32 (4 grayscales, or GS) to the highest PIQ level, most ML models encountered challenges in differentiating two facial classes (F10 and F14 indicated by red arrows shown on the right), while demonstrating decent discrimination for two other facial classes (F13 and F16 indicated by blue arrows). **(b)** All 16 facial classes (F01-F16) are shown here at the PIQ level of 32×32 (4 GS). F10 and F14 are outlined with red boxes, indicating facial classes that posed challenges even at the highest PIQ level, while F13 and F16 are outlined with blue boxes, signifying facial classes were easily discriminated. **(ci-ciii)** We examined the effects of facial conditions, gender, and age on the performance of the human groups (*i.e.*, G04-06) and the Best Model (*i.e.*, M05). While the human subjects exhibited varied performance across different facial expressions and facial angles, the Best Model showed fewer statistical differences across those facial conditions (*ci*). Unlike the NGB version (**Fig. 4cii-ciii**), both the humans and the Best Model showed statistically no different performances in terms of gender (*cii*) and age (*ciii*). Each bar represents the average and one standard deviation of 144 (*ci*), 360 (*cii*), and 225-270 (*ciii*) images, respectively (^*^*p* $<$ 0.05, ^**^*p* $<$ 0.01, ^***^*p* $<$ 0.001; Wilcoxon rank sum test). This research used datasets from 'The Open AI Dataset Project (AI-Hub, S. Korea)' with explicit permission from the original creators. All data information can be accessed through 'AI-Hub ([www.aihub.or.kr)](http://www.aihub.or.kr))' (Choi *et al.*, *arXiv*, 2021) ^[125]^. Human brain icon was obtained from Flaticon.com.


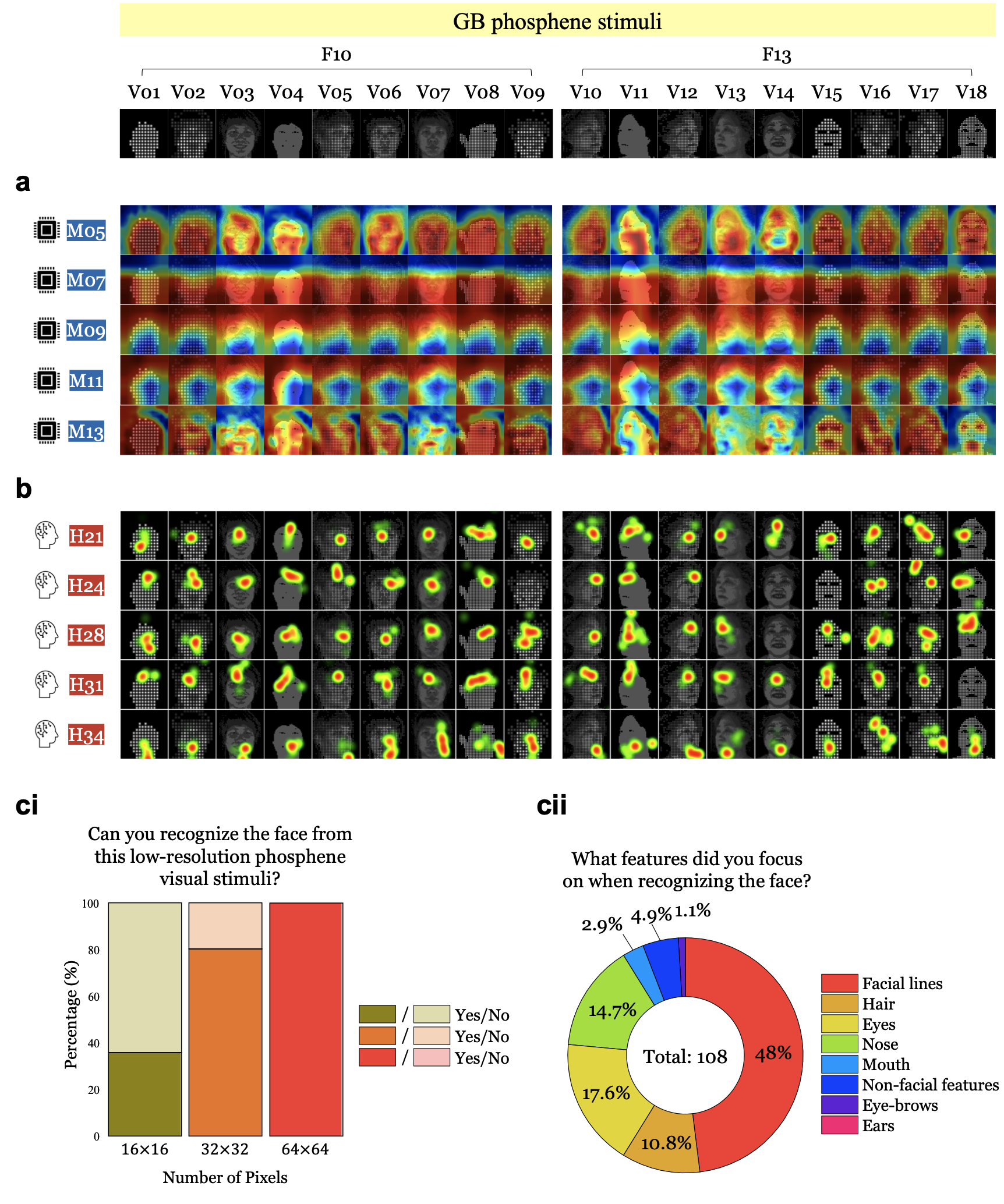


**Fig. S5. | The region-of-interest for facial recognition differs between deep learning (DL) models and humans for Gaussian-blurred (GB) version phosphene images. (a)** Gradient-weighted Class Activation Maps (Grad-CAMs) analysis was conducted for the four DL models (M05, M07, M09, and M11) along with one fine-tuned DL model (M13), using 18 sample phosphene images representing two facial classes (*i.e.*, V01-V09 for F10 and V10-V18 for F13). These samples covered various phosphene image quality (PIQ) levels and facial conditions. Notably, the Best Model (M05) consistently captured the facial features from the phosphene images better than the other models (M07, M09, M11, and M13). **(b)** The heat maps illustrating the relative duration of gaze area in five human subjects (H21, H24, H28, H31, and H34) were generated using Tobii Pro Fusion eye-tracker data. Across the participants, there was a consistent tendency to gaze more at the upper parts of the faces, as indicated by the red regions. **(ci-cii)** During the Post-test stage, survey responses were collected for each of the three different resolution levels with 4 grayscales (GS). First, 18 human subjects were asked if they recognized two faces represented with an array of phosphenes (36 responses in total for each resolution; 18 subjects × 2 facial classes). More than 60% of the subjects reported that they could not recognize faces from the 16×16 phosphenes, but more than three-quarters responded positively, starting from the 32×32 pixels (*ci*). Subsequently, those subjects were additionally asked about the visual features they had paid the most attention to (108 responses in total; 18 subjects × three resolutions × two facial classes). The human subjects had primarily focused on “facial lines” and “eyes” among various facial features (*cii*). This research used datasets from 'The Open AI Dataset Project (AI-Hub, S. Korea)' with explicit permission from the original creators. All data information can be accessed through 'AI-Hub ([www.aihub.or.kr)](http://www.aihub.or.kr))' (Choi *et al.*, *arXiv*, 2021) ^[125]^. Human brain icon was obtained from Flaticon.com.


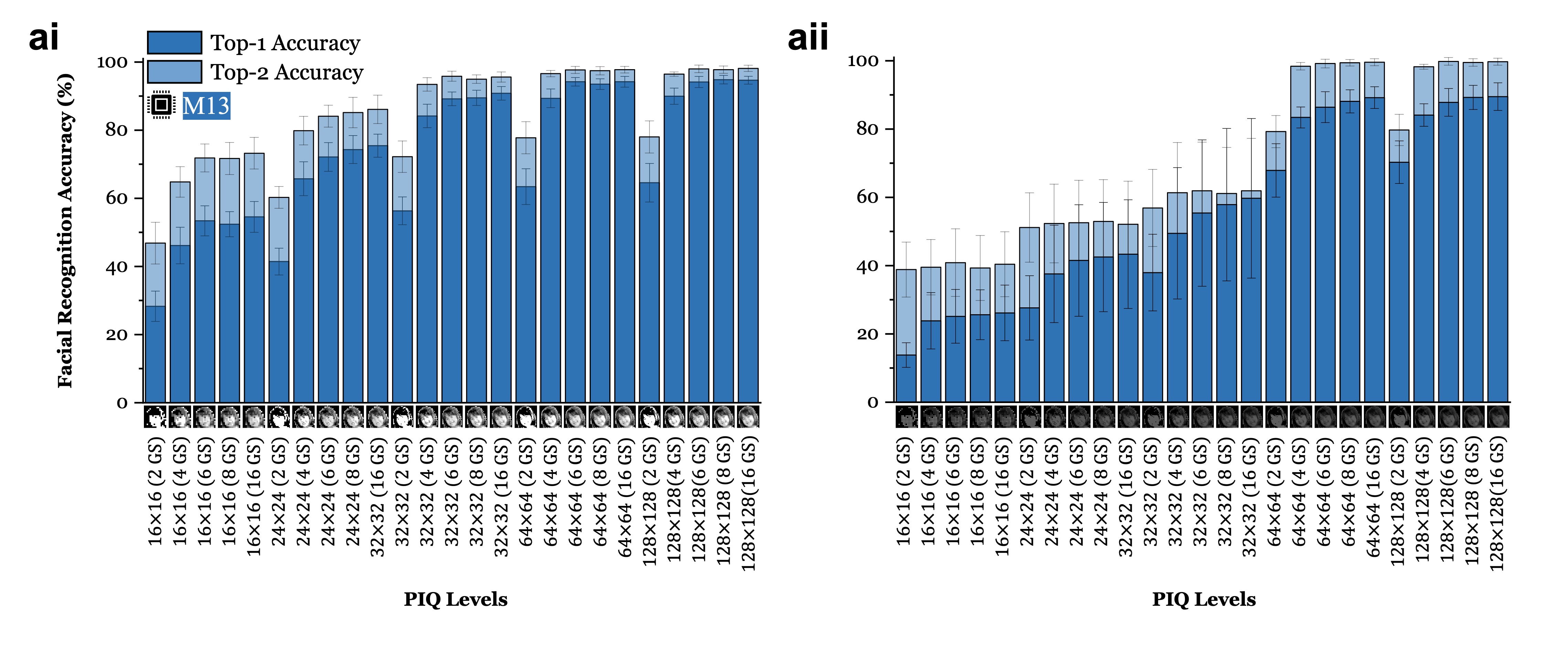


**Fig. S6. | The performances of a fine-tuned deep learning (DL) model (M13) were evaluated with varying phosphene image quality (PIQ) levels for both non-Gaussian-blurred (NGB) and Gaussian-blurred (GB) versions. (ai)** Both Top-1 and Top-2 facial recognition accuracies of M13, which is a fine-tuned version of M05 using NGB phosphene images, were higher than those of M05 for all the PIQ levels of NGB phosphene images (*compare* the plot with **Fig. 3ai**). **(aii)** Top-1 facial recognition accuracies of M13 were lower than those of M05 for the GB version (*compare* it with **Fig. S3ai**) since M13 was optimized for high-resolution during training stage and NGB phosphene images during fine-tuning stage (*see* Methods for details). Interestingly, however, the Top-2 facial recognition accuracies of M13 were higher than those of M05. This implies that fine-tuning using one type of phosphene (NGB in our case) may help to increase the facial recognition accuracies on the other type of phosphene (GB in our case) to some extent.


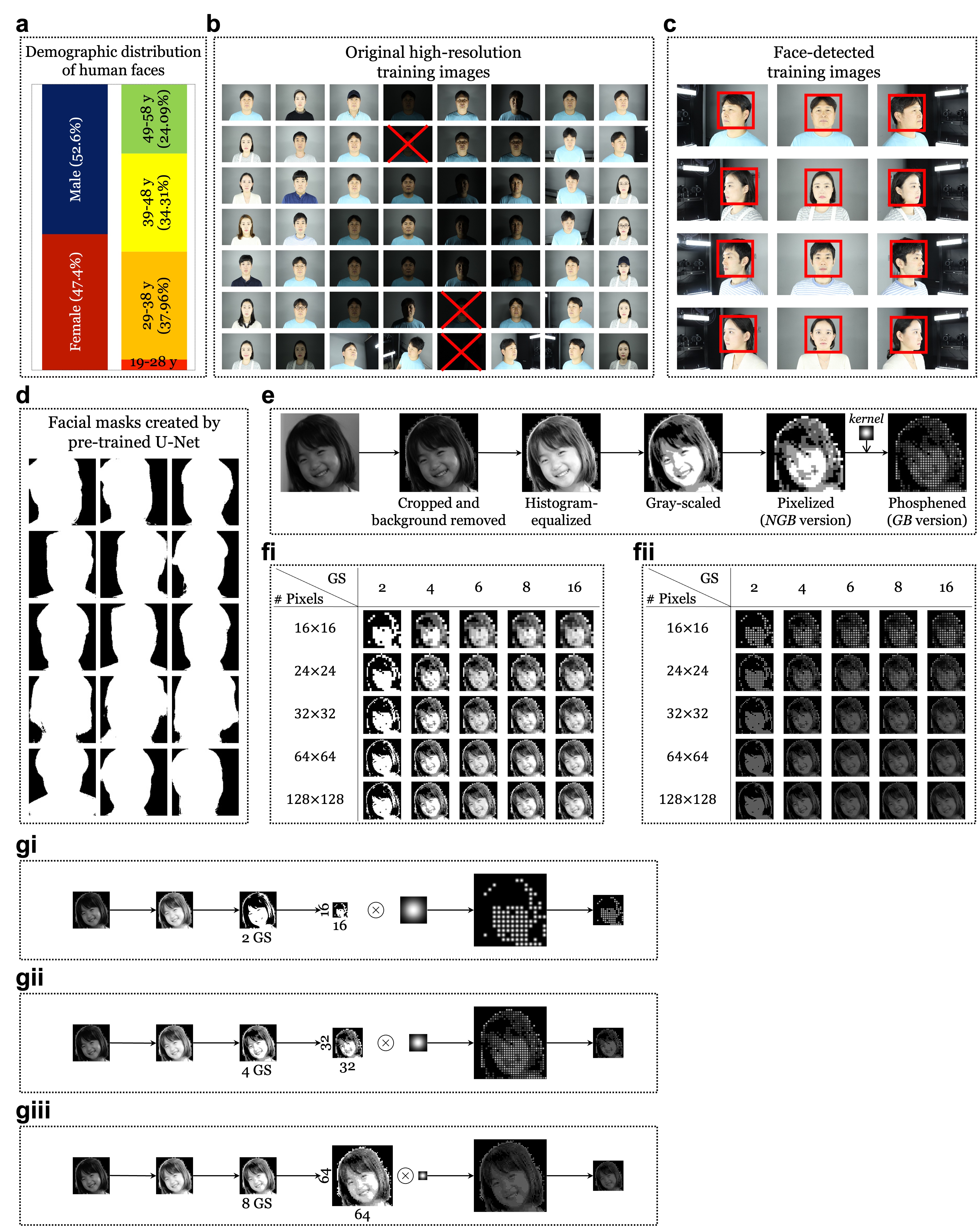


**Fig. S7. | Process of generating low-resolution phosphene images. (a)** The demographic distribution of the K-Face dataset (*see* Methods; y: years old) has almost equal gender division, and approximately 96.4% of individuals are aged over 29 years. **(b)** Some original high-resolution images were excluded due to poor lighting conditions, as indicated by red x marks. **(c)** Faces were detected using labeled and cropped into squares, delineated by red squares, with annotated labels denoting facial feature positions from the K-Face dataset. **(d)** Facial masks were generated using a pre-trained U-Net model to remove background noise and ensure a uniform background color. **(e)** The process of generating low-resolution phosphene images involved several steps, including cropping and background removal, histogram equalization, grayscale conversion, and pixelation for the non-Gaussian-blurred (NGB) phosphene images. Additionally, for Gaussian-blurred (GB) phosphene images, an extra step of overlaying phosphenes (*i.e.*, adding Gaussian kernel noise) was employed. **(fi-fii)** Phosphene images were generated with 25 phosphene image quality (PIQ) levels for each type of phosphene: NGB (*fi*) and GB (*fii*). **(gi-giii)** The GB phosphene images with varying resolution levels – 16×16 and 2 grayscales (GS) (*gi*), 32×32 and 4 GS (*gii*), and 64×64 and 8 GS (*giii*) – employed different kernel sizes: 16×16 (*gi*), 8×8 (*gii*), and 2×2 (*giii*), while maintaining consistent image dimensions. This research used datasets from 'The Open AI Dataset Project (AI-Hub, S. Korea)' with explicit permission from the original creators. All data information can be accessed through 'AI-Hub ([www.aihub.or.kr)](http://www.aihub.or.kr))' (Choi *et al.*, *arXiv*, 2021)^[125]^.

**
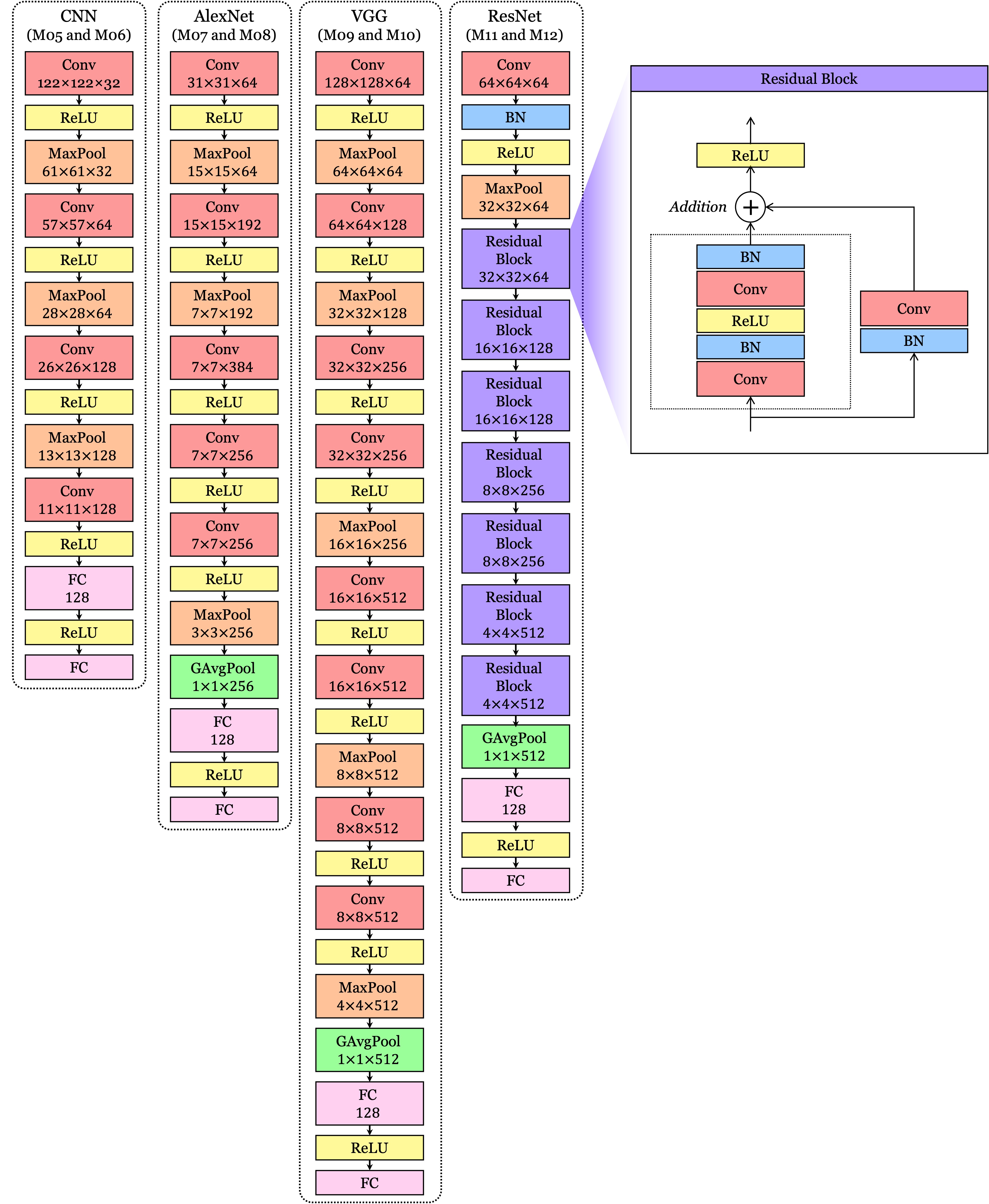
**

**Fig. S8. | Architectures of deep learning (DL) models.** The layer-wise architectures of the deep learning (DL) models examined in this paper are presented. Among all four models, CNN (*i.e.*, M05/M06) had the shallowest layer. We provided the output dimension (width × height × channel count) of an input image for various components, including convolutional (Conv), max pooling (MaxPool), global average pooling (GAvgPool), and Residual Block. It is worth noting that all models, similar to conventional machine learning (ML) models (*e.g.*, principal component analysis, or PCA), had 128 components (or artificial neurons) in the penultimate fully connected (FC) layer, and the number of artificial neurons in the last FC layer corresponded to the number of facial classes. The Residual Block from ResNet consisted of two Conv layers concatenated by a Conv identity layer that helps to preserve the features of the original input (ReLU: rectified linear unit function, BN: batch normalization).


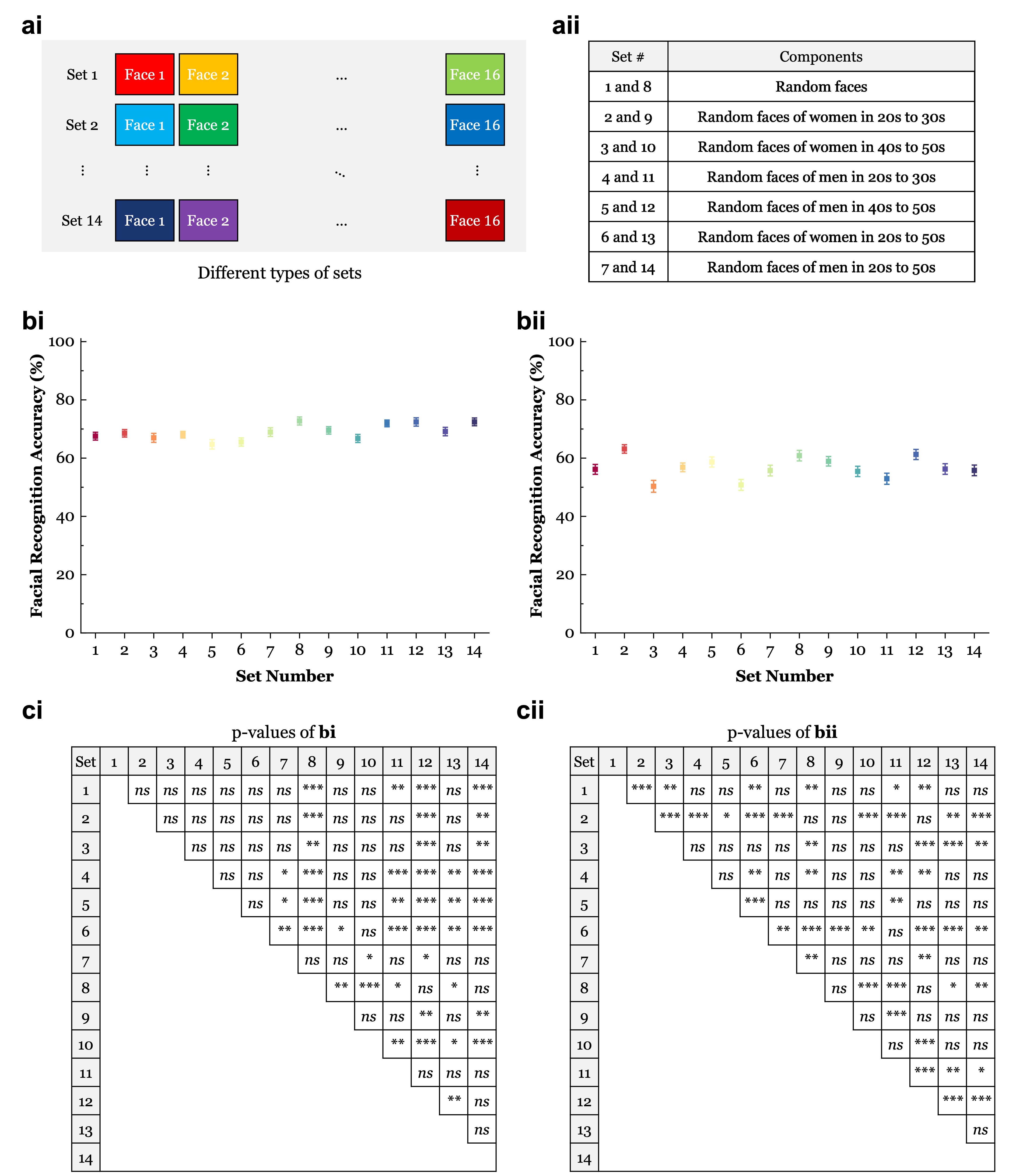


**Fig. S9. | No demographic effect on facial recognition accuracy was observed for the Best Model (M05). (a)** Fourteen different random test sets were created (*ai*), each with different demographic distributions (*aii*). Our initial hypothesis was that sex and age could affect the facial recognition accuracy of the Best Model; hence, we varied each set by changing the sex or age distribution. **(b)** However, there was no significant effect of set types on the average facial recognition accuracy for both the non-Gaussian-blurred (NGB; *bi*) and the Gaussian-blurred (GB; *bii*) versions for the Best Model. The error plots indicate standard errors of 25 phosphene image quality (PIQ) levels, 16 facial classes, and 9 facial conditions (*n* = 3,600). **(c)** The Best Model performances were consistent across different types of sets, and even when they were significantly different (indicated by ^*^, ^**^, or ^***^), it was not because one set had a demographic advantage over the other. In other words, even though we assumed that sets with mixed genders (*e.g.*, Set # 1) could help the model identify faces better in low-resolution phosphene images than the sets that included only one gender (*e.g.*, Set # 7), this was not the case. Also, the Best Model did not achieve better performances with phosphene face images that have a broader age gap (*e.g.*, 20s to 50s in Set # 7) than a relatively narrower age gap (*e.g.*, 20s to 30s in Set # 4). For instance, the Best Model accuracy for Set # 1 was similar to those of Sets # 2 through # 7, # 9, # 10, and # 14 in the NGB version (*ci*), and the accuracy for Set # 1 was not different from those of Sets # 4, # 5, # 7, # 9, # 10, # 13, and # 14 in the GB version (*cii*) (*ns*: not significant, ^*^*p* $<$ 0.05, ^**^*p* $<$ 0.01, ^***^*p* $<$ 0.001, Wilcoxon rank sum test).


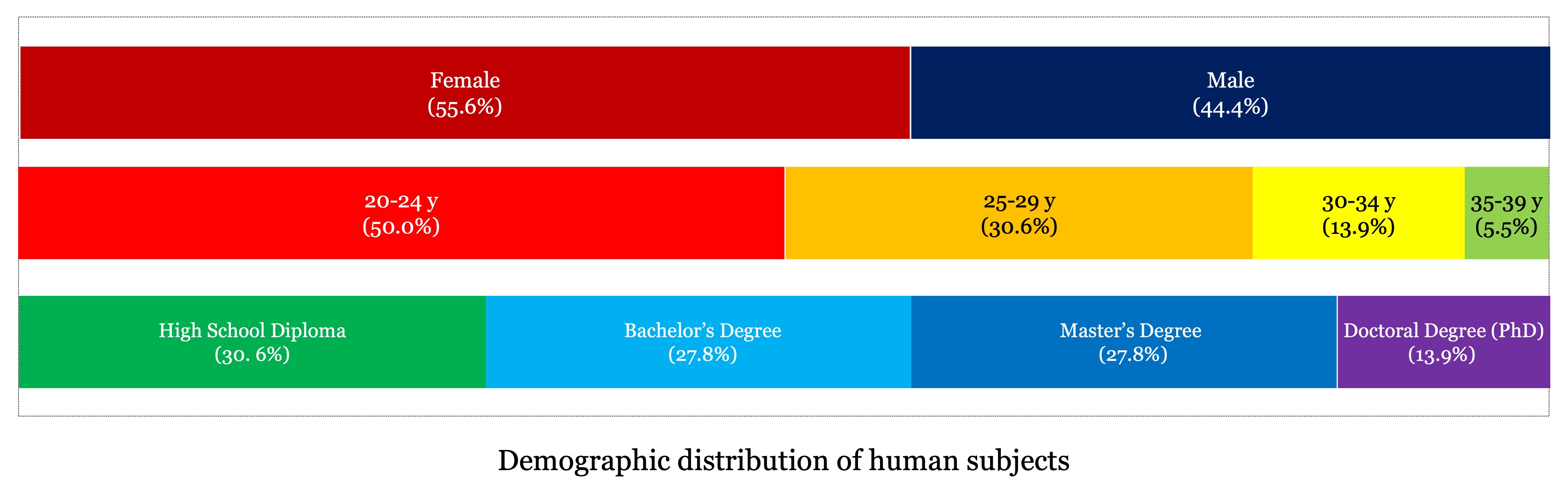


**Fig. S10. | The demographic distribution of human participants is presented in terms of sex, age, and education level.** We recruited 36 South Korean subjects, comprising 16 males and 20 females. Half of the participants were in the age group of 20-24 years old (y), followed by the age group of 25-29. None of the subjects were older than 40 at the time of psychophysical testing. In terms of education, all of them had at least a high school diploma.


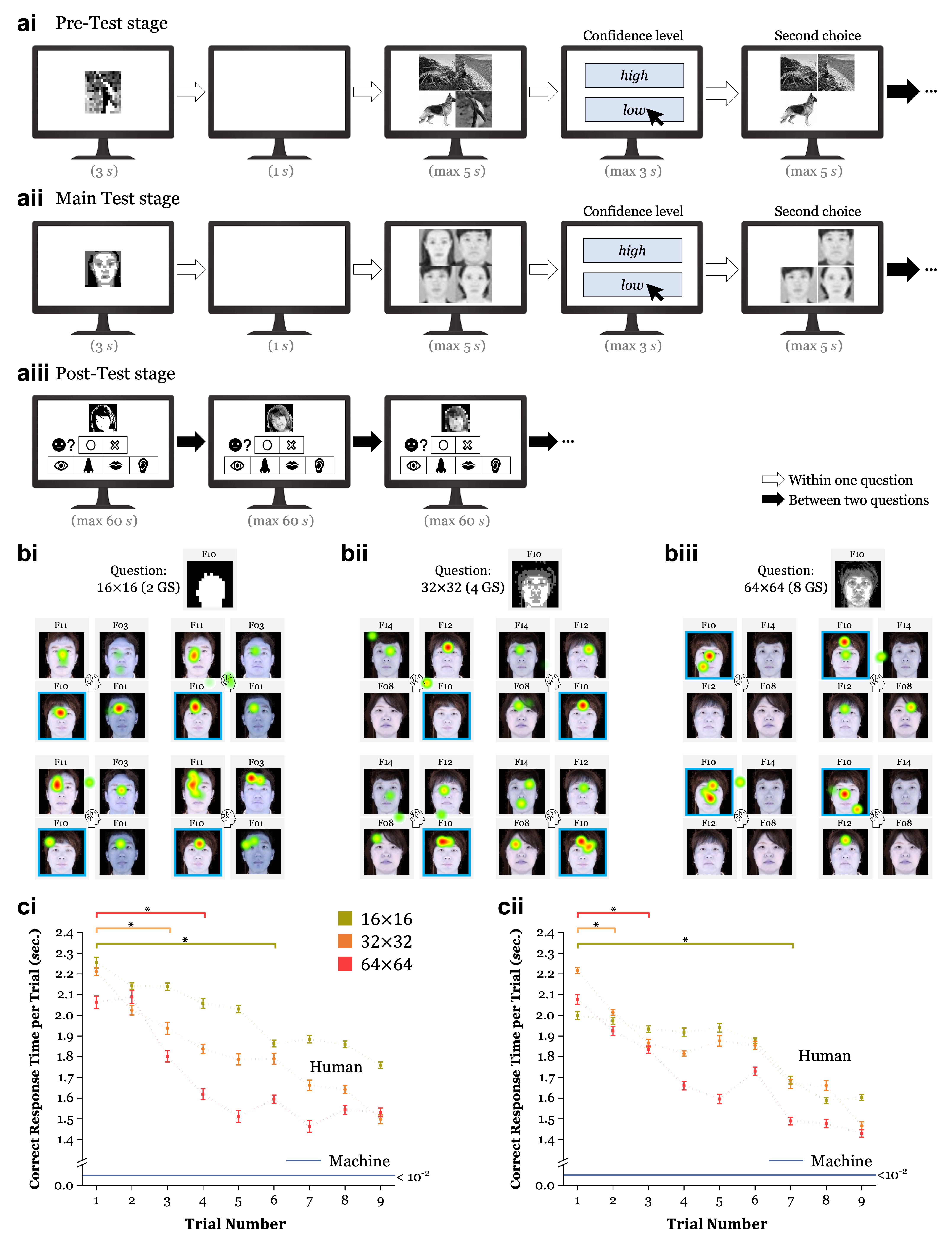


**Fig. S11. | Human psychophysical testing procedure, eye-tracking samples, and correct response time plot as a function of trial (or question) number. (a**) Each human participant underwent three stages of testing: **(ai)** Pre-test, **(aii)** Main test, and **(aiii)** Post-test. In each stage, the time duration of the individual screen is shown at the bottom of each screen (*s*: second). Non-facial images sampled from tiny ImageNet dataset were shown on the screens during the Pre-test stage. If a participant answered more than five questions correctly, they proceeded to the Main test session. During both the Pre-test and the Main-test stages, participants were allowed to select their second-best choice if they lacked confidence in their initial answer. The Post-test stage included six multiple-choice questions featuring low-resolution phosphene images (two facial classes; three resolution levels of 16×16, 32×32, and 64×64 with 4 grayscales (GS) for each facial class). The first part of each question asked participants whether they could recognize the human face from the low-resolution phosphene image. In the second part, they selected one of the eight options (facial lines, hair, eyes, eyebrows, nose, mouth, ears, and non-facial features) representing the features they focused on when identifying the face from the given low-resolution phosphene image. **(b)** Eye-tracking data samples are shown for each question at three resolution levels: **(bi)** 16×16 (2 GS), **(bii)** 32×32 (4 GS), and **(biii)** 64×64 (8 GS). Relative duration heat maps are shown for four different human subjects in the three resolution levels. Correct facial class answers are outlined with blue boxes. As the resolution level increased, each human subject could quickly fixate their eyes on the correct facial class, indicated by a red heat map only on the correct facial class for the resolution level of 64×64 (8 GS). **(c)** The correct response time for facial recognition accuracy was measured for **(ci)** non-Gaussian-blurred (NGB) and **(cii)** Gaussian-blurred (GB) versions. In both types of phosphenes, the correct response time per trial exceeded 1.4 seconds for all resolution levels in humans, which is tremendously longer than the inference computation time of the ML models (< 10 milliseconds). Error bars represent standard errors of 5 GS levels, 16 facial classes, and 2 to 4 facial conditions (*n* = 160 to *n* = 320) (^*^*p* $<$ 0.05, ^**^*p* $<$ 0.01, ^***^*p* $<$ 0.001, Wilcoxon rank sum test). This research used datasets from 'The Open AI Dataset Project (AI-Hub, S. Korea)' with explicit permission from the original creators. All data information can be accessed through 'AI-Hub ([www.aihub.or.kr)](http://www.aihub.or.kr))' (Choi *et al.*, *arXiv*, 2021)^[125]^.


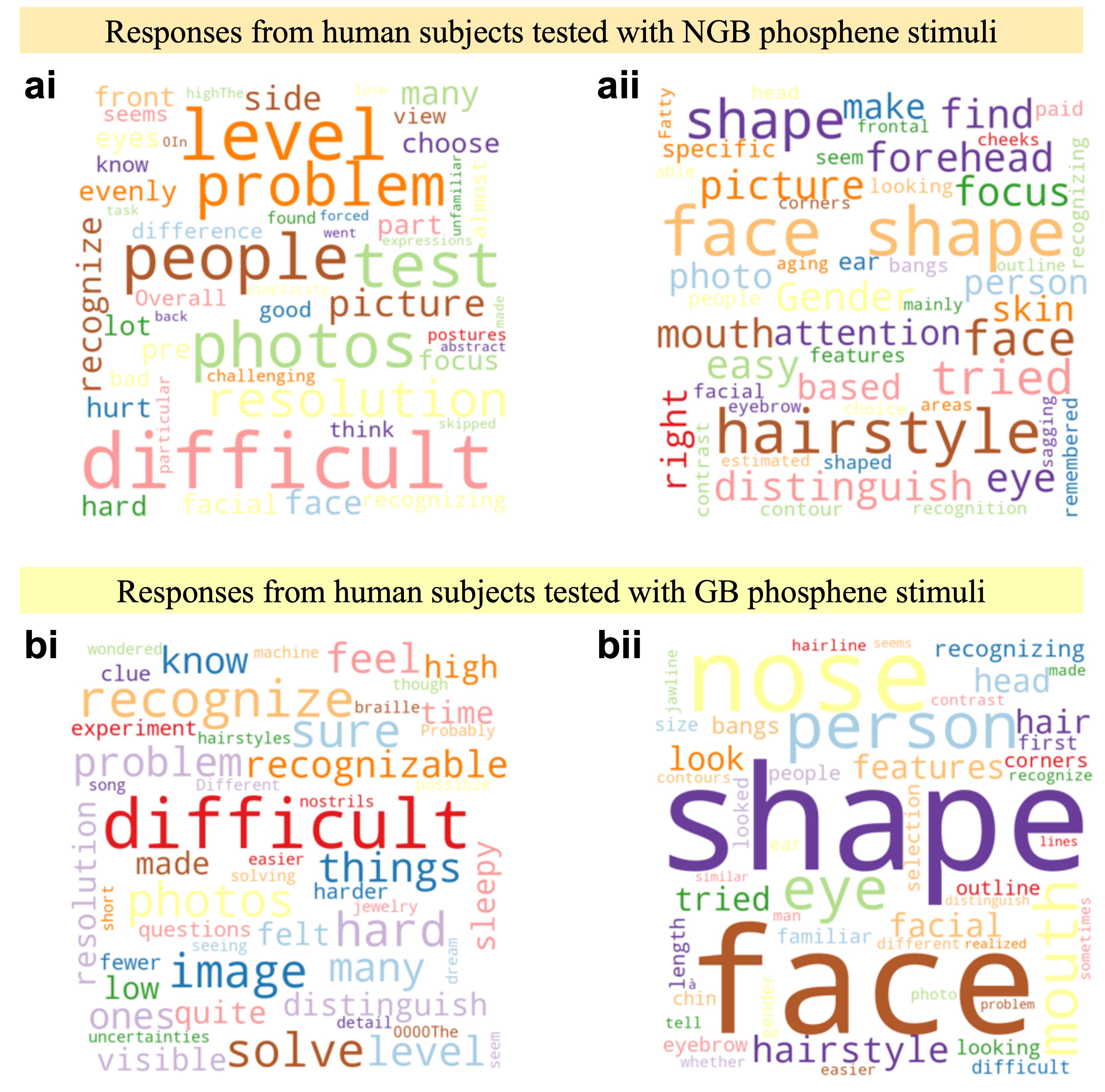


**Fig. S12. | Word clouds are generated from the Post-test essay responses (Table S1) collected from 36 human participants. (ai)** The responses of 18 human subjects tested with non-Gaussian-blurred (NGB) phosphene face images are visualized in a form of word clouds, with the word “difficult” appearing most frequently when asked the difficulty level since they were not used to viewing faces in the forms of phosphene arrays. However, it should be noted that the size of the word “difficult” is similar to the size of the other words that also frequently appeared in the responses, such as “level,” “problem,” “people,” and “photos,” suggesting that these word combinations were commonly used across human subjects. **(aii)** The Top-5 large-sized nouns that emerged in the word cloud generated from responses to essay questions regarding where participants predominantly looked while attempting to recognize faces rendered on low-resolution phosphene images were “face shape,” “hairstyle,” “shape,” “eye,” and “forehead,” consistent with the responses to short-answer questions (**Figs. 5cii**). This indicates these larger facial features and “eyes” were more helpful cues for recognizing faces in NGB phosphene images than comparably smaller facial features, such as “mouth” and “ears.” **(bi)** Same as ***ai*** but for the responses of 18 human subjects tested with Gaussian-blurred (GB) phosphene face images. In line with the NGB version, the word “difficult” appeared most frequently in their responses regarding the difficulty level because they were completely new to artificial vision. However, the size of the word “difficult” is significantly larger than the other follow-up frequently appeared words, such as “recognizable” and “problem” in comparison to the NGB version. This suggests that the GB facial phosphenes were indeed more challenging for human subjects to recognize than the NGB facial phosphenes. **(bii)** Same as ***aii*** but for the responses of the subjects tested with GB phosphene face images. The Top-5 large-sized nouns that appeared in the word cloud were “shape,” “face,” “nose,” “mouth,” and “eye,” consistent with the responses to short-answer questions (**Figs. S5cii**). This result is also consistent with the NGB version, where the “face shape” seemed to be the most crucial feature in recognition. However, compared to the NGB version, smaller facial features, such as “mouth,” could be beneficial for recognizing GB facial phosphenes.

**Supplementary Movie**


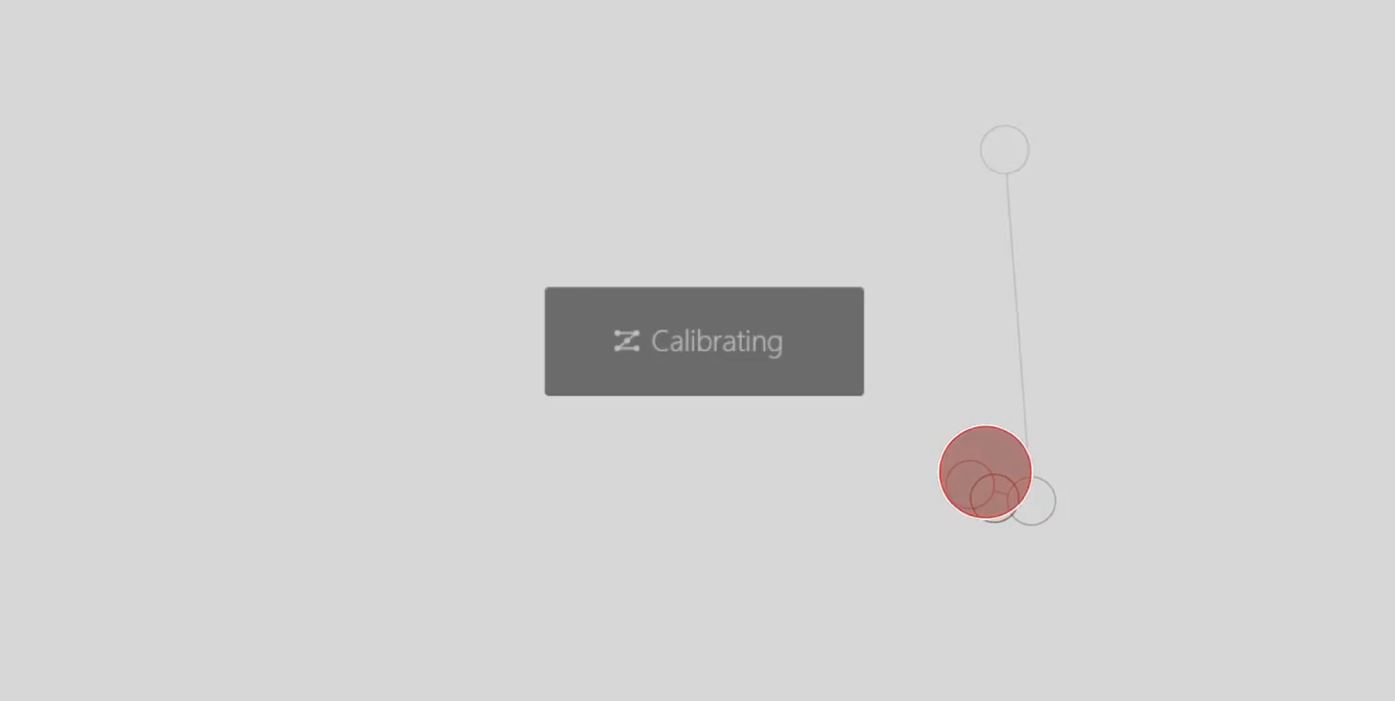


**Supplementary Movie S1. | A sample video clip (double speed) displays the eye tracking results of the human psychophysical experiment.** This video features a cropped view of a real-time experiment conducted on one human subject (H02), who was tested with non-Gaussian-blurred (NGB) phosphene images. Throughout the video, the viewer can observe eye tracking data. The experiment commences with the Pre-test stage, lasting until 1:35. Subsequently, the first two questions of the Main-test stage are presented from 1:52 to 2:16, followed by questions # 321 and # 322 at 2:18 and 2:29, respectively. The final five questions are displayed starting from 2:48 onwards.

**Supplementary Table**

**Table S1. | Post-test essay responses from human subjects were collected during breaks and after the experiment.** Participants were asked two essay questions: 1. What is the perceived difficulty level of the test you were given? 2. Where did you mostly look at while trying to recognize faces rendered on low-resolution phosphene images? All the responses were initially written in Korean, and can be obtained by emailing one of the authors. For convenience of reviewers and readers, the English version translated by DeepL (<https://www.deepl.com/translator>) is presented below.

| **Human subject #** | **Level of difficulty** | **Focused facial features** |
| --- | --- | --- |
| **H01** | I found the difficulty level very challenging.  In particular, the postures and facial expressions were unfamiliar to me. | When recognizing people, I paid attention to their hairstyle, collar, etc. to remember their features.  As I went backwards, I remembered the features of each person and the shape of their face, making it less difficult than it was in the beginning. |
| **H02** | The difficulty level is very high  The simplicity of the task made me lose focus the further back I went.  There were many abstract problems that I was forced to take pictures of or that I skipped over. | You seem to be looking for things in your head or in the background |
| **H03** | In the Pre-test, the pixelated (degraded) photos of the same part (or similar parts) were evenly distributed, but when the test entered the Main test, the photos were evenly composed to resemble frontal photos of people, but it was very difficult to choose due to the variety of people shown (smiling faces, side views, etc.). |  |
| **H04** | - The problem is hard.  - There are so many problems. | - I picked it based on contour, head, etc. |
| **H05** | Difficulty: Hard | Recognition Spotlight: Gender, Face Shape, and Bangs |
| **H06** | A lot of difficulty | Attention to hair style (bangs in a bun, etc.). Note the face shape as well. Unrecognizable in black and white with no contrast. Something recognizable in the background of the photo other than the face shapes. |
| **H07** | Some of the pictures were so hard to recognize as human faces. | The rest of the pictures I overcame facial contours, hairstyles (M-shaped forehead, hairpins), etc. |
| **H08** | Toward the end (about an hour in), I lost focus and my eyes hurt. My eyes hurt too much and I can't concentrate. | I started with human face recognition, which was number one in the order. Pixel, resolution, face shape, left-right asymmetry, and hairstyle were compared and selected, and a frontal image was given as a choice, and the example had multiple angles, so I had to make a prediction and make a choice. Then there were facial expressions (corners of the mouth, mouth shape, smiles, etc.) Jawline, face shape, hairline, and nostrils were crucial to distinguish, and eye and eyebrow shape were easy to distinguish. Skin aging and sagging were easy to distinguish (aging and older people were easy to find) (dark, sagging skin, drooping skin, nasolabial folds). Fatty cheeks were relatively light and easy to pick out. Curved or dimpled areas, fatty cheeks. |
| **H09** | Difficulty: Difficult  1. difficulty with side-to-front analogies  2. difficulty recognizing facial expressions | Where did you focus your attention when recognizing:  1. roundness and elongation of the face  2. proportionality of forehead, pharynx  3. eye corners, wrinkles  4. gender-specific contours  5. forehead-areas covered by hairstyle |
| **H10** | There seems to be a big difference between bad and good resolution (difficulty is higher in bad resolution). | Recognition mainly sees the outline of the face and 2 ears. The photos are mostly similar, which is confusing. |
| **H11** | *<Not answered>* | *<Not answered>* |
| **H12** | - It seems to be more difficult, and you only see the same people, so you choose based on their mood or feelings. | - You chose based on hairstyle or face shape. |
| **H13** | There were many photos that were almost indistinguishable, | but even with the low quality, I was able to distinguish 1. face shape 2. estimated age 3. male/female for the most part. The biggest distinction was face shape, and I saw two main types. Round faces. There were many moon-shaped faces. We conducted the experiment by judging the face shape by looking at the two lengths of the face. Recognition ranking: Face shape -> Estimated age -> Gender There seemed to be a big difference between frontal and side views. |
| **H14** | It was so difficult that I almost took a picture of it. | I tried to match based on similarities in face shape, eye, nose, and mouth position, and hairstyle.  When I tried to focus on specific parts to find the right picture, it was hard to choose.  Later, I tried to memorize the overall outline to find the right picture.  I found this method to be ineffective because I was getting sleepy and losing focus.  As the picture in question was repeated, I felt that the difficulty became a little easier as I remembered the person.  I tried to categorize them as male vs. female and young vs. old. |
| **H15** | Low resolution was difficult to recognize because it only showed the position of the nose | Noted hairstyle (forehead shape), face shape, and eye color. |
| **H16** | The difficulty level was increased compared to the Pre-test. | I mainly paid attention to the hairstyle (whether it was a kama, ear covering, perm), mouth shape (+eyebrow shape) (+ear shape) (+fleshiness) Initially, I tried to find the person in the photo, but later on, I learned and was able to predict who it was. |
| **H17** | 1. it was harder than I thought it would be. | 2. Most of the time I seem to focus on the face shape or hairstyle, or where the contrast will be. |
| **H18** | - | - |
| **H19** | Overall, it's very difficult to match the side and front view, and some of them I can barely recognize the face shape. I don't know if that helps, because sometimes I just have to take a picture of it. | tried to make it gender-specific and focused on the size of the cheekbones, nose shape, and hairstyle. |
| **H20** | - The problem was difficult.  - I felt like I was solving the same problem. |  |
| **H21** | Different hairstyles and jewelry made it easier to distinguish. Photos with low resolution or no visible nostrils were difficult to distinguish. | The facial skeleton was used to identify whether it was a man or a woman. |
| **H22** | It was too difficult... The time given to recognize was too short... (Probably, it felt more so because of the difficulty.) | I mainly recognized the head shape/eyebrow shape (contrast). |
| **H23** |  | - If the hair shape (length, garma, etc.) was prominent, it was easier to recognize even if the face was hard to see.  - As I became more familiar with the faces of the characters, I made more confident selections compared to the beginning.  - In the case of facial expressions, the different face shapes, eye and nose shapes, and so on, made the selection more often unsure or unsuccessful.  - In the case of the familiar faces, the person's face was sometimes the first thing that came to mind when the caricature was shown (before the subsequent selection photo), even at low quality |
| **H24** | The difficulty level is quite high. | Start by looking at the outline of the face (jawline, neck length, hairline, etc.) and if you can't tell, look for features and contrast them. (shading, jawline, etc.). As you go backwards, you get more and more familiar with the person's face, and you can see the contours that you couldn't see before. |
| **H25** | It was so difficult that I wondered if it was even possible for a machine to recognize that level of detail. | I tried to distinguish between the eyes and the corners of the mouth, but sometimes the answer candidates with the corners of the mouth up were the only ones with the corners of the mouth down, so I didn't think that was enough. I realized that I could pay attention to hair, eyebrows, and facial lines, but there are still a lot of similarities, so I'm not sure if I can do it with confidence. |
| **H26** | Difficulty Difficult. Too many uncertainties and hard to recognize. Too many things I'm not sure about, but some things I'm sure about. The song is very sleepy. I feel like I'm in a dream. Too sleepy. I feel like I'm seeing things even though I'm not sure. | There are too many people with similar features, face shape, hairline. (I tried to look at the nose at first, but it didn't work, so I looked at the whole frame. I'm bored with the same problem. I'm sleepy, but it's difficult.) |
| **H27** | Other than the braille image, the other images seem to be easy. | When recognizing, it seems to look at hairstyle 🡪 nose 🡪 in that order. Once the age and gender are predicted, it seems to be convincing just by looking at the hairstyle. |
| **H28** |  | The hairstyle alone helped me distinguish them, but if they all had the same hairstyle, it would have been very difficult to tell them apart (+ body type). |
| **H29** | The first time I tried the experiment, I found it very difficult, but as I got used to it, I was able to solve some pictures. If you have a very unique face, you will be more confident. It's harder to solve photos with a bad angle. | I solved it by looking at the shape of the face (oval, egg-shaped). Some questions I got confused by the pixelated photos, and others I solved because the other three out of four choices were too different, so I realized it was either an OX problem or a subjective one. |
| **H30** | 30-40% recognizable. 60% don't even know who they are (difficulty) The more questions you solve, the more fatigued your eyes become and the more difficult it is to recognize. | When recognizing them, I checked the facial features (eye size, shape, etc.) and face shape (egg, elongated, chubby, etc.) + the vibe...? I can't express it, but I also look at hair style, etc. Gender first. Determine whether the person is foreign or chubby through their face shape. The shape and size of the eyes and nose. If there is a gender-restricted person in the prophecy, it can make the choice easier, even if it is difficult to recognize. |
| **H31** | It was more difficult with photos that were completely black and white with no contrast and fewer dots. | When recognizing, I focused on the shape of the bangs and chin and looked closely at the ear and nose positions. I distinguished between people with similar chins by the length of their noses in relation to the position of their ears. As I kept seeing the same people over and over again, I found myself remembering the actual man in the image rather than remembering the specific outlines of the dotted lines on his face. I labeled the dotted images in my head as "the guy with no bangs," "the guy with the pointy chin," "the lady with the full head of hair," and so on, trying to find the specific person. |
| **H32** | I feel that the difficulty level is quite higher than I thought. I think there are about 40% of the problems that even I, a non-disabled person, am not sure about. It was more difficult when showing the side view. I solved the binary image almost as soon as I took it. Reducing the channel of the image too small gave fewer clues and made it harder to solve. The experiment was longer than expected, and the number of questions was high, which led to fatigue, which may have a negative effect on the correct answer rate. Overall: Difficult! | I focused on the contours of the face when recognizing, and the hairstyle was also a big clue. |
| **H33** | The more I try, the less I can focus.  I don't know what to do with the pixelated ones in low resolution, I don't know their gender, I don't have a clue.  There are too many problems. | I focus on the shape of the bangs, the shape of the face. |
| **H34** | The hard ones were very hard, and the recognizable ones were recognizable. | I looked at the head, the outline of the face, and the mouth of the eyes. |
| **H35** | It was difficult to find similar photos because the features were hard to recognize if they were not clearly visible or had large pixels. | I tried to remember the features of each person (bangs, ears, mouth shape, nostril size, eyebrows, etc.) and then tried to find them. |
| **H36** | At 2 levels of contrast, I don't think you can tell the difference, no matter how good the resolution. Looks like it should be at least level 4. The number of people is so small that I don't know if I'm memorizing or recognizing them because I keep seeing them. Overall, I struggled a lot. I can only think of a handful of times when I was 100% sure of my choice. | Seemed to see a lot of hairstyles. Especially with the bangs. |
